# Genotypic and Phenotypic Diversity among Human Isolates of Akkermansia muciniphila

**DOI:** 10.1128/mBio.00478-21

**Published:** 2021-05-18

**Authors:** Bradford Becken, Lauren Davey, Dustin R. Middleton, Katherine D. Mueller, Agastya Sharma, Zachary C. Holmes, Eric Dallow, Brenna Remick, Gregory M. Barton, Lawrence A. David, Jessica R. McCann, Sarah C. Armstrong, Per Malkus, Raphael H. Valdivia

**Affiliations:** a Department of Molecular Genetics and Microbiology, Duke University, Durham, North Carolina, USA; b Department of Pediatrics, Duke University Hospital, Durham, North Carolina, USA; c Division of Immunology & Pathogenesis, Department of Molecular and Cell Biology, University of California, Berkeley, California, USA; University of Hawaii at Manoa

**Keywords:** *Verrucomicrobia*, comparative genomics, phylogroups, microbiome, mucin, assimilatory sulfur reduction (ASR), adolescent obesity, phylogenetic analysis

## Abstract

The mucophilic anaerobic bacterium Akkermansia muciniphila is a prominent member of the gastrointestinal (GI) microbiota and the only known species of the *Verrucomicrobia* phylum in the mammalian gut. A high prevalence of *A. muciniphila* in adult humans is associated with leanness and a lower risk for the development of obesity and diabetes. Four distinct *A. muciniphila* phylogenetic groups have been described, but little is known about their relative abundance in humans or how they impact human metabolic health. In this study, we isolated and characterized 71 new *A. muciniphila* strains from a cohort of children and adolescents undergoing treatment for obesity. Based on genomic and phenotypic analysis of these strains, we found several phylogroup-specific phenotypes that may impact the colonization of the GI tract or modulate host functions, such as oxygen tolerance, adherence to epithelial cells, iron and sulfur metabolism, and bacterial aggregation. In antibiotic-treated mice, phylogroups AmIV and AmII outcompeted AmI strains. In children and adolescents, AmI strains were most prominent, but we observed high variance in *A. muciniphila* abundance and single phylogroup dominance, with phylogroup switching occurring in a small subset of patients. Overall, these results highlight that the ecological principles determining which *A. muciniphila* phylogroup predominates in humans are complex and that *A. muciniphila* strain genetic and phenotypic diversity may represent an important variable that should be taken into account when making inferences as to this microbe’s impact on its host’s health.

## INTRODUCTION

The gastrointestinal (GI) microbiota comprises a complex community of bacteria, fungi, and archaea that significantly influence the metabolic and immunological health of their human and animal hosts (recently reviewed in reference 1). The global obesity epidemic has focused attention on the role that the microbiota plays in regulating energy acquisition and inflammation and how these activities impact the development of metabolic disease and diabetes (2). Western-style diets, in particular, lead to microbiotas of lower taxonomical diversity and metabolic capacity, which in turn, enhance the risk for developing inflammatory disorders such as diabetes, obesity, and cardiovascular disease (3–5).

Akkermansia muciniphila is a Gram-negative anaerobic bacterium of the phylum *Verrucomicrobia* that can use GI mucins as a sole carbon and nitrogen source (6, 7). *A. muciniphila* has attracted considerable attention because an increased abundance of *Akkermansia* in the GI tract correlates with many positive human health outcomes, including protection from obesity, diabetes, and metabolic disease (8–11). Indeed, lean individuals show an increased representation of *Verrucomicrobia* in their fecal microbiomes as assessed by 16S rRNA gene profiling (9). *A. muciniphila* is prevalent in the colon and has been reported to comprise between 1 and 4% of the total bacteria in healthy adult fecal samples (12). In mice, repeated administration of *A. muciniphila* ameliorates the impact of high-fat diets in inducing obesity and strengthens the function of the GI epithelial barrier though the activation of Toll-like receptor 2 (TLR2) (13, 14). In proof-of-concept trials in humans, administration of live or pasteurized *A. muciniphila* was sufficient to improve insulin sensitivity and reduced insulinemia (15).

Two recent pangenomic studies of *Akkermansia*, including a comparison of 35 *A. muciniphila* genomes reconstructed from metagenomic sequences (16) and 39 *A. muciniphila* isolated strains (17), led to the identification of four distinct phylogroups (clades AmI to IV). An analysis of the genomes of representative members of these phylogroups revealed phylogroup-specific functions such as the ability of AmII strains to synthesize corrin rings (16) and thus potentially outcompete other phylogroups when levels of vitamin B_12_ precursors in the GI tract are scarce. Given that multiple *A. muciniphila* phylogroups are found in humans, we asked what is the genomic and phenotypic diversity of *A. muciniphila* isolated from children, with the long-term goal of determining if there are correlates between strain and phylogroup abundance and specific health outcomes. To begin to address these questions, we used fecal samples collected by the Pediatric Obesity Microbiome and Metabolome Study (POMMS) (18) to isolate 71 unique strains of *A. muciniphila*. Participants included adolescents with healthy weight at a single time point only and adolescents with obesity at baseline and then at 6 months following a behavioral lifestyle intervention. A subsample of the participants with obesity additionally received pharmacotherapy or bariatric surgery during the study period. We performed a detailed genotypic and phenotypic analysis of these isolates and determined that POMMS *A. muciniphila* strains belonged to three main phylogroups/clades. Several *in vitro* traits, such as growth rates in mucin, resistance to ambient oxygen, self-aggregation, and binding to epithelial surfaces were linked to specific phylogroups. Furthermore, we determined that even though specific phylogroups displayed GI tract colonization advantages in antibiotic-treated mice, the parameters influencing *Akkermansia* colonization in humans are more complex.

## RESULTS

### The relative abundance of *A. muciniphila* in fecal samples is highly variable in a cohort of children with obesity.

**FIG 1:**
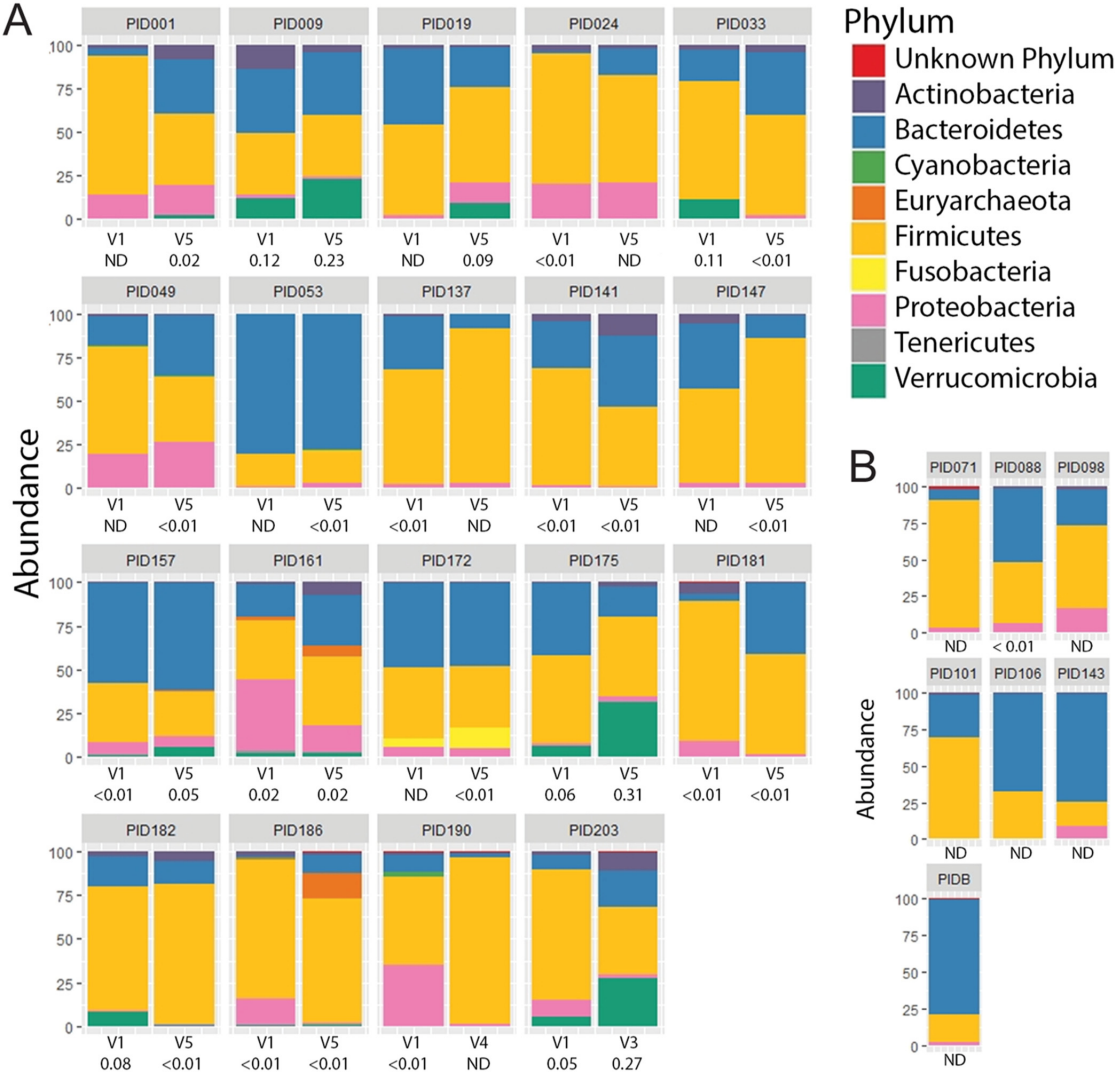
Relative abundance of *Verrucomicrobia* in stool samples of children with obesity. (A and B) Phylum-level assessment of the composition of bacterial communities in fecal samples derived from children with obesity before and 6 months after undergoing treatment for weight loss (A) and representative children of healthy weight (B) enrolled in the same study. The identity and relative representation of bacteria within each stool sample were determined by amplification and sequencing of the 16S RNA locus. The normalized fraction (%) of *Verrucomicrobia* is provided under the visit (V) number. ND, not detected.

To survey the diversity of *A. muciniphila* strains present among healthy children and those with obesity, we used fecal samples that had been collected before and after various interventions aimed at decreasing their body mass index (BMI) (18). We selected 5 lean controls (Z-score adjusted BMI, or zBMI, –0.99 to 0.41) and 36 children with extreme obesity (zBMI, –1.63 to 3.18 > 95th percentile) (Table S1). For healthy lean control children, a single stool sample was obtained at the time of enrollment. For the majority of the cohort with obesity (35/36), we used samples collected at baseline and at the end of the study (6 months). We enriched for mucolytic bacteria directly from frozen fecal material by serial passage in liquid medium with gastric porcine mucin as the sole carbon and nitrogen source and then isolated single colonies on mucin agar plates. Bacterial colonies were purified to homogeneity and typed by sequencing the V3-4 region of the 16S rRNA locus. Overall, we cultured 71 strains of *A. muciniphila* from 35 children and 1 adult. In parallel, we performed a 16S rRNA-based survey of the bacterial communities present in selected fecal samples for which we had baseline and at least one additional visit (Fig. 1 and Table S2). A phylum-level analysis indicated that the relative abundance of *Verrucomicrobia* ranged from undetectable to 31% of total bacteria (Table S2).

10.1128/mBio.00478-21.1 TABLE S1 Summary of pediatric donors described in this study. Download Table S1, XLSX file, 0.01 MB. Copyright © 2021 Becken et al. 2021 Becken et al. https://creativecommons.org/licenses/by/4.0/ This content is distributed under the terms of the Creative Commons Attribution 4.0 International license.

10.1128/mBio.00478-21.2 TABLE S2 ASV tables and relative abundance and composition of microbial communities in POMMS samples. Download Table S2, XLSX file, 0.02 MB. Copyright © 2021 Becken et al. 2021 Becken et al. https://creativecommons.org/licenses/by/4.0/ This content is distributed under the terms of the Creative Commons Attribution 4.0 International license.

### *A. muciniphila* clinical isolates are phenotypically diverse.

Previous analysis of microbial metagenomes indicated that there is significant diversity among *A. muciniphila* strains (19). To begin to address if this genetic diversity correlates with traits of relevance to the colonization and health of the human host, we performed a range of phenotypic tests that have been associated with *Akkermansia* biology, including variations in (i) growth rates, (ii) the ability to form bacterial aggregates, (iii) adherence to epithelial surfaces, (iv) the generation of short-chain fatty acids (SCFA) during mucin fermentation, (v) sensitivity to oxidative stress, and (vi) activation of Toll-like receptors (TLRs).

**FIG 2:**
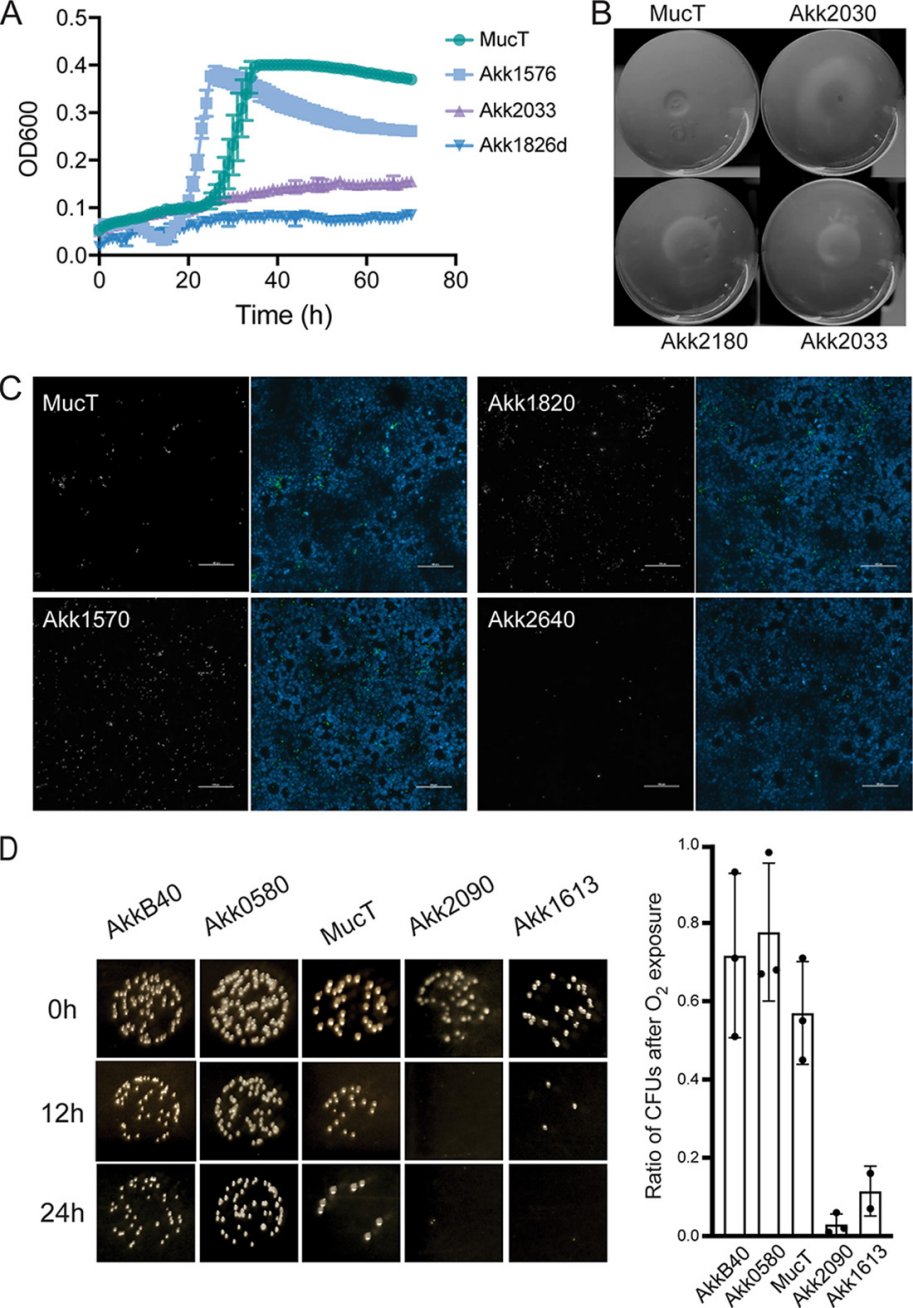
*A. muciniphila* human isolates display variance in phenotypes relevant to gastrointestinal colonization. (A) *A. muciniphila* strains display distinct growth rates in porcine gastric mucin. The growth rates of *A. muciniphila* isolates were monitored in liquid medium over a period of 96 h. Representative examples of fast and slow growers are shown in relation to the typed *A. muciniphila* strain Muc^T^. (B) *A. muciniphila* strains agglutinate. Selected strains shown as examples of rapid sedimentation of *A. muciniphila* grown in mucin medium. (C) *A. muciniphila* strains vary in their ability to attach to epithelial surfaces. Adhesion of selected *A. muciniphila* strains to HT29 colonic epithelial cells was assessed by immunofluorescence microscopy. HT29 nuclei were detected with Hoechst (blue) and bacteria with polyclonal anti-*Akkermansia* antiserum (white, left panels; green, right panels). Scale bar = 100 μm. (D) *A. muciniphila* strains vary in their tolerance to ambient oxygen. Strains grown in BHI supplemented with mucin were exposed to ambient oxygen for 0, 12, or 24 h on BHI-mucin agar plates followed by outgrowth under anaerobic conditions. Oxygen sensitivity was assessed by enumerating CFU on agar plates.

**Growth rates.** The growth rates among individual strains were monitored under anaerobic conditions in a semidefined synthetic medium consisting of glucose, *N*-acetylglucosamine, soy peptone, and threonine. *A. muciniphila* strains displayed doubling times ranging from 1.2 to 13 h. These differences were more pronounced when gastric porcine mucin was the sole carbon and nitrogen source, with doubling times ranging from 0.3 to >20 h (Fig. 2A and Table S3).

10.1128/mBio.00478-21.3 TABLE S3 Summary of *A. muciniphila* isolates and phylogroups and their phenotypes. Download Table S3, XLSX file, 0.02 MB. Copyright © 2021 Becken et al. 2021 Becken et al. https://creativecommons.org/licenses/by/4.0/ This content is distributed under the terms of the Creative Commons Attribution 4.0 International license.

**Aggregation**. Some *A. muciniphila* isolates, unlike the reference strain *A. muciniphila* Muc^T^, readily sedimented when grown in liquid culture media (Fig. 2B). We measured the extent to which strains aggregated by monitoring the changes in the optical density in samples obtained from the surface of culture tubes that had been grown in mucin medium (Table S3).

**Adherence to epithelial cells.** We determined if there are strain-level differences in *A. muciniphila* isolates for their ability to attach to intestinal epithelial cells. Binding was assessed using HT29-MTX colonic epithelial cells grown on microtiter plates for 7 days past confluence, a stage at which they start secreting mucins. As a control for nonspecific binding to biotic surfaces, we used bovine serum albumin (BSA)-coated plates. Adherence was monitored either microscopically or by following the outgrowth of *A. muciniphila* after addition of synthetic medium to wells. As previously reported, *A. muciniphila* strain Muc^T^ bound to human colonic cells (20), albeit at low levels. Our *A. muciniphila* isolates displayed a broad range of binding affinities to HT29 monolayers (Fig. 2C, Fig. S1A and B, Table S3) compared to BSA-coated plates. Strains also differed in their binding to BSA-coated plates alone (Fig. S1C).

10.1128/mBio.00478-21.7 FIG S1 *A. muciniphila* binding to HT29 colonic epithelial cell and protein-coated plated. Adherence of *A. muciniphila* strains isolated from patients enrolled in the POMMS to HT29 colonic epithelial cell- or BSA-coated plates. Bacteria were incubated with HT29-MTX cell- or BSA-coated plates and extensively washed, and binding was indirectly evaluated by adding synthetic medium to the wells and measuring the optical density at 600 nm after 48 to 72 h. (A) Binding is provided as the ratio of the OD_600_ HT29-MTX to BSA-coated plates. (B and C) The nonnormalized OD_600_ units for HT29-MT (B) or BSA-coated (C) plates are provided for reference. Bars represent the mean and standard deviation of 3 technical replicates from 3 independent experiments. Download FIG S1, TIF file, 2.7 MB. Copyright © 2021 Becken et al. 2021 Becken et al. https://creativecommons.org/licenses/by/4.0/ This content is distributed under the terms of the Creative Commons Attribution 4.0 International license.

**Mucin fermentation.** The major end products of mucin metabolism by *A. muciniphila* are acetate and propionate, with minor amounts of succinate and 1,2-propanediol (16, 21). The ratio of acetate to propionate generated is influenced by how simple sugars are metabolized and the availability of vitamin B_12_ to activate methylmalonyl-coA and generate propionate (16). We measured SCFAs produced by the various *Akkermansia* strains after growth in mucin by gas chromatography. All *A. muciniphila* isolates produced acetate/propionate at a ratio of 1.21 to 1.47 after reaching the stationary phase of growth in 0.5% mucin medium, which is in the range of what has been reported for the *A. muciniphila* Muc^T^ strain (21).

**Oxygen sensitivity.** Although *A. muciniphila* is an anaerobe, its growth can be stimulated by low levels of oxygen (22). We predicted that the relative tolerance of *A. muciniphila* to oxygen may impact its abundance near epithelial surfaces or its resistance to oxidative stress during GI inflammation. We tested the sensitivity of *A. muciniphila* strains to oxygen by exposing agar plates to ambient air for 12, 18, and 24 h before returning the plates to anaerobic conditions (Fig. 2D). We determined that there is a range of responses, with some strains being fairly tolerant to prolonged exposure to ambient oxygen (∼60% survival at 24 h), while others were extremely sensitive (<0.01% survival at 12 h) (Table S3).

**FIG 3:**
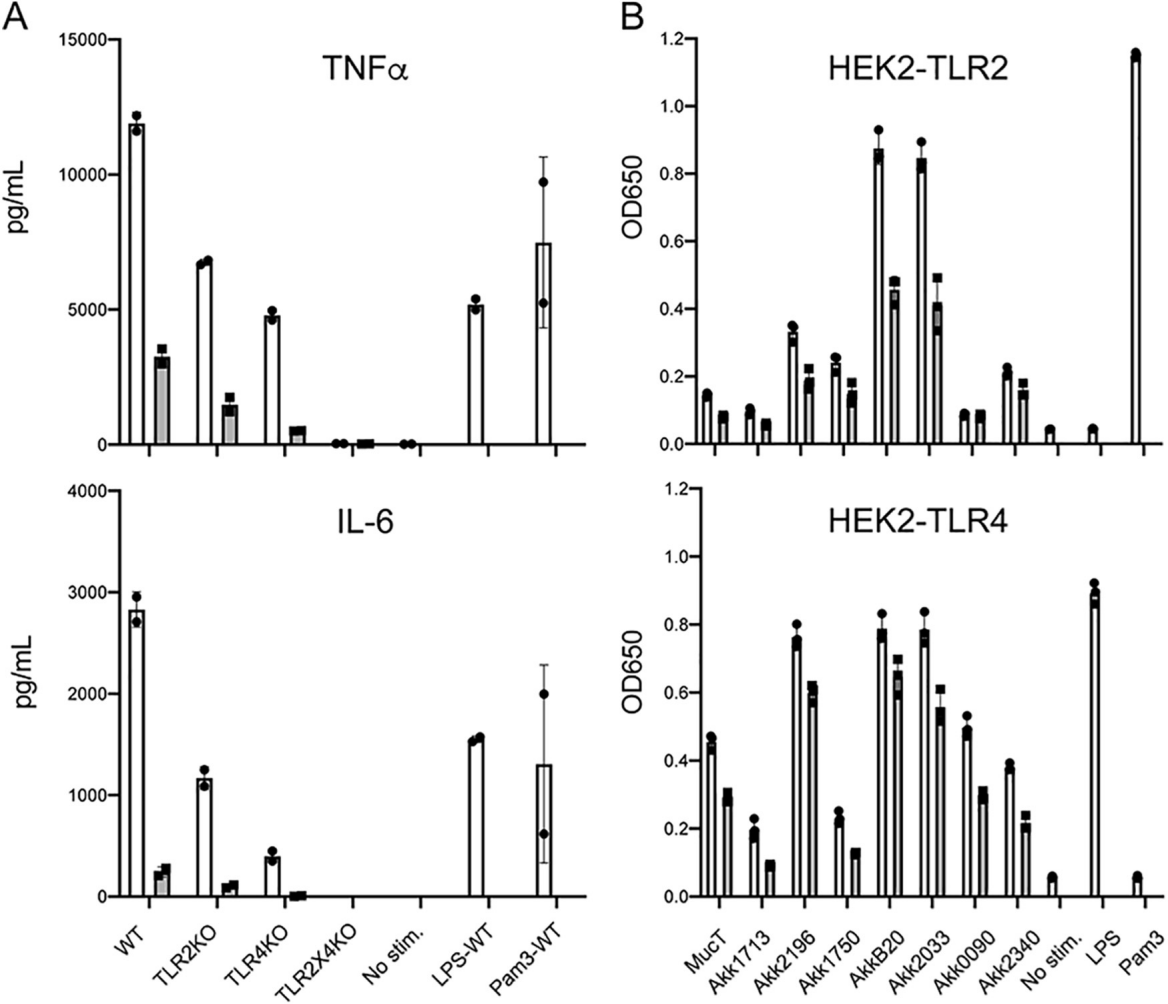
Activation of TLR2 and TLR4 by *A. muciniphila* isolates. (A) TLR2 and TLR4 are the main microbial sensors required for the recognition of *A. muciniphila*. Bone marrow-derived macrophages from the selected *tlr* knockout mice were incubated with *A. muciniphila* Muc^T^ at a ratio of 5 (white bars) or 0.5 (gray bars) per BMDM for 6 h, followed by measuring TNF-α or IL-6 as readouts of activation. BMDM activation only occurs in cells expressing either TLR4 or TLR2, with TLR4 displaying a lower threshold for *A. muciniphila* stimulation. (B) *A. muciniphila* strains vary in their ability to activate TLR2 and TLR4. Selected strains grown on mucin medium were incubated with HEK293 reporter cell lines expressing either TLR2 or TLR4 at a ratio of 5 or 1 bacteria/cell (white and gray bars, respectively). Stimulation of TLR in these cell lines was determined by assessing the processing of a colorimetric substrate of secreted of alkaline phosphatase.

**Activation of innate immune sensors.** Human monocytes are activated by exposure to *A. muciniphila* Muc^T^ (23), and TLR2-dependent recognition is required for signal transduction events that strengthen barrier function in the GI tract (24). We first tested to be determine the relevant TLRs involved in the recognition of *A. muciniphila* by stimulating bone marrow-derived macrophages (BMDM) from wild-type C57BL/6J mice and from *tlr4*, *tlr2 tlr4*, *unc93b1*, and *tlr2 tlr4 unc93b1* knockout mice (25). Unc93b1^−/−^ mice are defective for the transport of TLRs that sense nucleic acids as well as the expression of TLR5, which recognizes flagellin, at the cell surface (26, 27). BMDM were incubated with *A. muciniphila* Muc^T^ at a ratio of 0.5 or 5 bacteria per cell for 6 h, and the secretion of the cytokine tumor necrosis factor alpha (TNF-α) and interleukin-6 (IL-6) was assessed (Fig. 3A). At the lowest dose of *Akkermansia*, the response of BMDM was exclusively dependent on TLR4. TLR2-dependent activation was only apparent when bacterial loads were increased by 10-fold. Consistent with previous findings (23), additional TLRs were not required for immune activation of mouse BMDMs.

We next used HEK-TLR reporter cell lines to test the ability of *A. muciniphila* isolates to specifically activate TLR2 and TLR4. We used cell lines expressing TLR4 or TLR2 and its coreceptors TLR1 and TLR6, which respond to known TLR2 ligands (28). *A. muciniphila* strains were grown in mucin or synthetic medium and incubated with reporter cell lines expressing TLR2 or TLR4 for 16 h at a ratio of 1 or 5 bacteria per HEK-TLR cell, and levels of TLR-dependent secreted alkaline phosphatase (sAP) were measured 16 h poststimulation (Fig. 3B and Table S3). A subset of strains induced TLR2 or TLR4 activation consistently above or below the mean of all *A. muciniphila* isolates tested (Table S3 and Fig. S2). The induction of TLR2 and TLR4 reporters was higher for bacteria grown in synthetic medium compared to mucin medium (Fig. S2).

10.1128/mBio.00478-21.8 FIG S2 *A. muciniphila* activation of HEK293-TLR reporter cell lines. (A to D) *A. muciniphila* strains were grown on mucin or synthetic medium and incubated with HEK293 reporter cell lines expressing either TLR1/2/6 (A and B) or TLR4 (C and D). Activation of the TLR in these cell lines was determined by assessing the secretion of alkaline phosphatase into culture supernatants. Data are reported as the normalized fold induction over the mean OD value of the entire population. Bars represent the mean and standard deviation of 3 technical replicates from 3 independent experiments. Download FIG S2, TIF file, 0.9 MB. Copyright © 2021 Becken et al. 2021 Becken et al. https://creativecommons.org/licenses/by/4.0/ This content is distributed under the terms of the Creative Commons Attribution 4.0 International license.

### Comparative genomics suggest that phylogroup-specific genetic determinants regulate *A. muciniphila* replication in mucin and aerotolerance.

**FIG 4:**
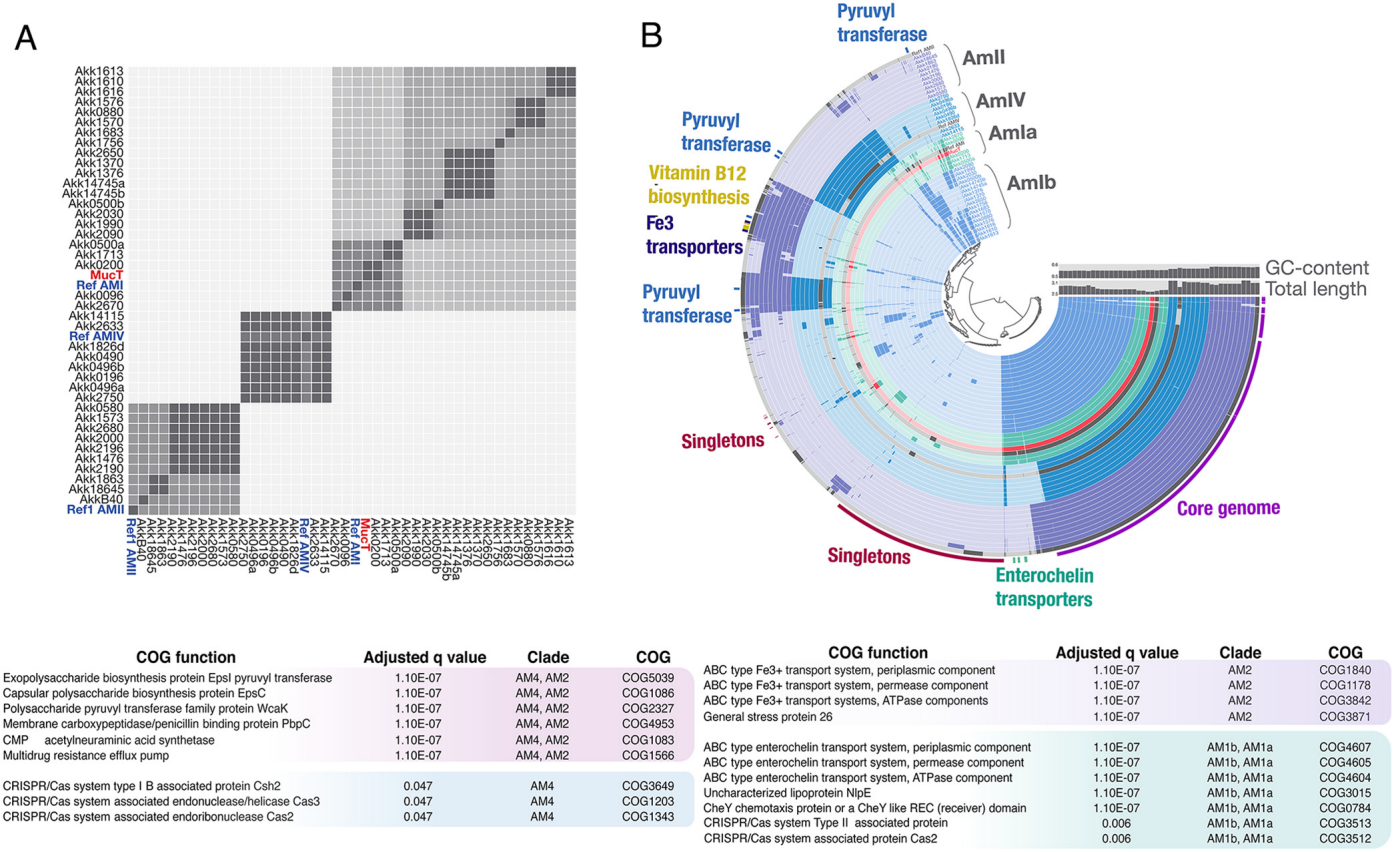
*A. muciniphila* isolates from children belong to three different phylogroups. (A) Whole-genome comparison of 40 *A. muciniphila* strains. The average nucleotide identity (ANI) was calculated at 96% identity using Anvi’o and PyANI. Genomes for previously published strains (16, 17) belonging to each *Akkermansia* phylogroup were included as controls (blue), as well as the type-strain Muc^T^ (red). Complete genomes were assembled from PacBio reads. Note that phylogroup AMI can be subdivided into two subtypes at 96% ANI threshold. (B) Circle phylogram of new *A. muciniphila* strains. The graph displays the pangenome of 40 sequenced isolates, 3 reference genomes (gray), and the type strain Muc^T^ (red). The phylogram is clustered based on gene frequency and displays gene cluster presence/absence for each genome. Selected phylogroup-specific gene groups are highlighted to show their distribution, including vitamin B_12_ biosynthetic gene groups, putative enterochelin transporters, Fe^3+^ transporters, and capsule genes (labeled as pyruvyl transferases). (C) Gene set enrichment analysis of *A. muciniphila* phylogroups. Anvi’o was used to identify COG functions associated with specific phylogroups. The selected COGs were detected in all isolates belonging to a given phylogroup(s) and in none of the isolates belong to other phylogroups. The adjusted *q* value shows the significance of the enrichment between the function and the associated phylogroup corrected for multiple testing.

Of the four *A. muciniphila* phylogroups (clades AmI to IV) the most studied strain (Muc^T^) is a representative of phylogroup AmI (16, 17). To assess the distribution of *A. muciniphila* phylogroups in the POMMS cohort, we sequenced the genomes of 43 isolates. A comparative analysis of these genomes indicated that we have representatives of phylogroups AmI, AmII, and AmIV, but not AmIII (Fig. 4). We designed phylogroup-specific primers (Table S4) to genotype the isolates whose genomes had not been sequenced and determined that AmI (41/71) was almost twice as prevalent as AmII (22/71) and AmIV (8/71). Based on whole-genome comparisons of AmI members, we propose that AmI can be further subdivided into two related subclades (Ia and Ib) at a threshold of 96% average nucleotide identity (ANI). Genomes ranged in size from 2.6 to 3.3 Mb, and phylogroups AmII and AmIV were consistently larger than the AmI genomes (Fig. 4A and B and Table S3).

10.1128/mBio.00478-21.4 TABLE S4 Primers used for the identification of *A. muciniphila* phylogroups and quality control for specificity. Download Table S4, XLSX file, 0.01 MB. Copyright © 2021 Becken et al. 2021 Becken et al. https://creativecommons.org/licenses/by/4.0/ This content is distributed under the terms of the Creative Commons Attribution 4.0 International license.

**FIG 5:**
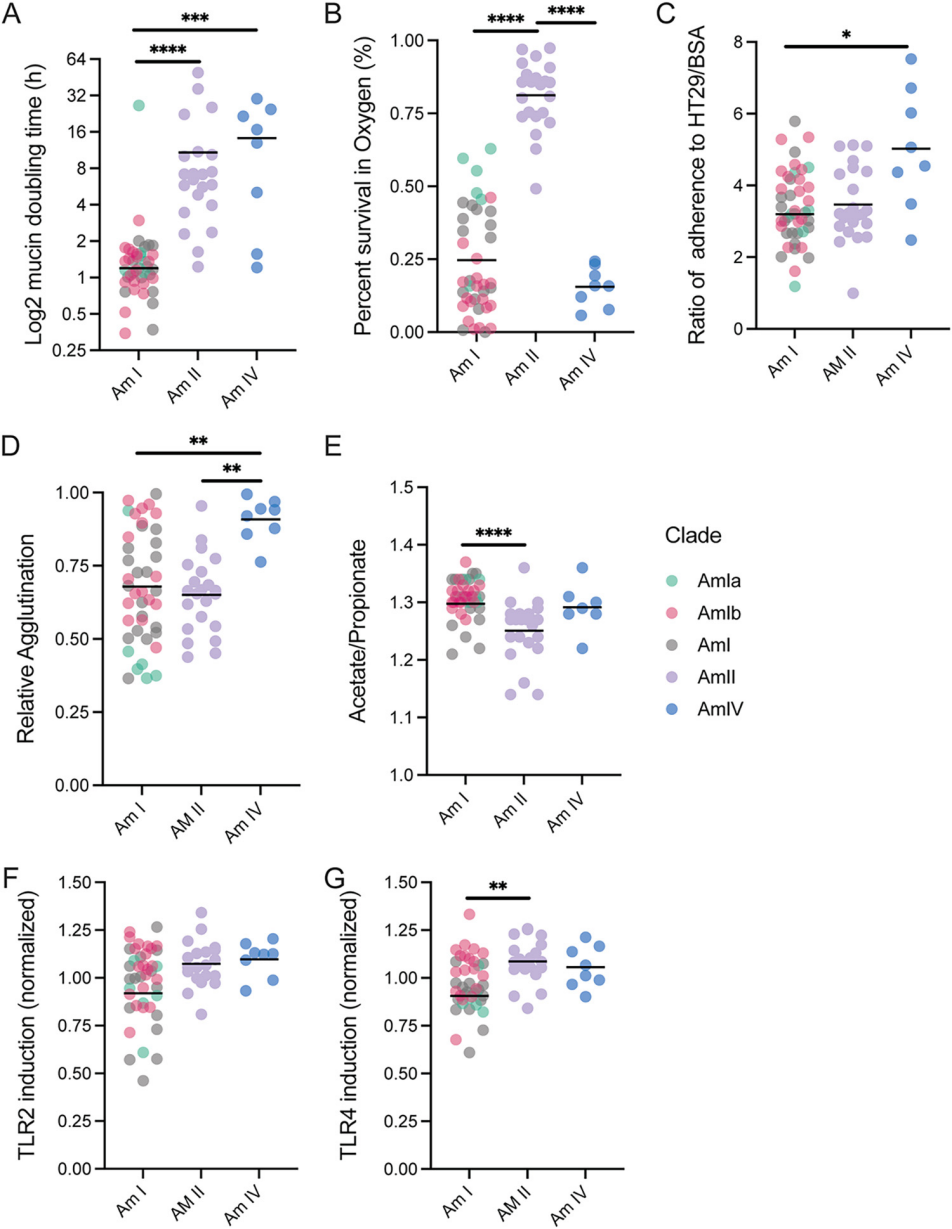
*A. muciniphila* strains display phylogroup-dependent and independent phenotypes. (A to G) Distribution of *A. muciniphila* (A) doubling times in mucin medium, (B) resistance to ambient oxygen on agar plates, (C) adherence, (D) agglutination, (E) mucin fermentation, (F) TLR2 activation, and (G) TLR4 activation. Each symbol represents a strain. AmI strains that were not subtyped into AmIa or AmIb are shown as gray dots. Details are available in Table S3. *P* values were calculated using Kruskal-Wallis tests followed by Dunn’s multiple-comparison tests. *, *P* < 0.05; **, *P* < 0.01; ****, *P* < 0.0001.

We determined that several phenotypes segregated by phylogroup (Fig. 5). AmI strains displayed rapid doubling times, while most members of AmII and AmIV grew slowly (Fig. 5A). Strains also differed in their sensitivity to ambient oxygen, with strains of AmII being resistant and those of AmIV very sensitive (Fig. 5B). Differential oxygen sensitivity was also observed within phylogroup AmI, with AmIb strains being highly sensitive to exposure to air, while AmIa strains displayed intermediate resistance. AmIV strains had higher adherence to epithelial cells and displayed a greater propensity to aggregate when grown in mucin medium (Fig. 5C and D). We also saw a small drop in the ratio of acetate/propionate fermentation end products of mucin fermentation for AmII strains (Fig. 5E), which we postulate is because they synthesize vitamin B_12_ (16) and hence generate more propionate as vitamin B_12_ in the growth medium becomes limiting.

For the stimulation of HEK-TLR reporter cell lines, AmII and AmIV strains were more stimulatory for both TLR2 and TLR4 than AmI strains (Fig. 5F and G), but it is unclear if this is simply a reflection of their enhanced binding properties to cell surfaces. AmI strains displayed a broad range of activation of TLR reporter cell lines, and while there was a trend for AmIb strains to be more stimulatory, particularly for TLR4, the differences did not reach statistical significance given the relatively low number of AmIa isolates in our strain collection.

To identify genes that may contribute to these phenotypes, we analyzed the pangenome of our *A. muciniphila* strains and identified 4,982 total gene clusters, with 1,647 core gene clusters found in all genomes and 506 gene clusters found only in single genomes (Fig. 4A). We found several phylogroup-specific gene groups that may contribute to phenotypic variation (Fig. 4B and C and Table S3). For example, all members of phylogroups AmII and AmIV were predicted to encode distinct capsule and exopolysaccharide genes that were absent in AmI strains. These included putative capsular polysaccharide (CPS) biosynthesis proteins EpsC and EpsI, a CMP-*N*-acetylneuraminic acid synthetase, and a capsule modifying enzyme, polysaccharide pyruvyl transferase WcaK (29). The AmI isolates have a single *Cps* locus, while AmII and AmIV genomes contain two additional loci that are largely conserved among these phylogroups and map to similar regions in the chromosome. This suggests common capsule types are present in the phylogroup AmII and IV strains. Phylogroup AmIV also codes for DltB, an enzyme typically involved in modification of lipoteichoic acid in Gram-positive bacteria but that can also modify lipopolysaccharides in some Gram-negative bacteria (30). Conversely, members of phylogroup AmI had additional chemotaxis genes, cytochrome c biosynthetic genes, and code for a quality control sensor protein for outer membrane biogenesis, NlpE (31).

The phylogroups also displayed differences in iron acquisition systems. Anaerobic conditions favor reduced ferrous iron (Fe^2+^), and aerobic conditions favor the oxidized ferric iron (Fe^3+^) (32). Although all genomes had a ferrous iron transport system consisting of FeoAB genes (Amuc_1088, Amuc_1089, and Amuc_1090 in Muc^T^), phylogroups AmI and AmII encoded additional mechanisms to acquire ferric iron. Members of AmI had multiple enterochelin transporter gene groups, suggesting that they might use siderophores to scavenge ferric iron. Members of phylogroup AmII also have predicted ferric iron transporters, although these appear to be distinct from the gene groups in phylogroup AmI. In contrast, phylogroup AmIV lacks canonical mechanisms for ferric iron acquisition. Phylogroup AmIV is highly sensitive to ambient oxygen, and it is plausible that defects in iron acquisition or the absence of the oxidative stress protection associated with siderophores (33, 34) may contribute to this phenotype. Additional contributors to differences in oxygen sensitivity include a LexA repressor, indicative of an SOS system, that is present in all phylogroup AmI and AmII strains.

The *A. muciniphila* strains were predicted to encode approximately 27 glycoside hydrolase (GH) enzyme families, but they varied in the abundance of GH families among the phylogroups, particularly in AmIV genomes (Fig. 3B). GH97 enzymes, which comprise glycoamylases such as Bacteroides thetaiotaomicron SusB (35), were detected in all strains except for AmIV. Similarly, AmIV genomes had fewer GH110 enzymes, a group of galactosidases capable of cleaving blood group B antigens (36). Conversely, AmIV strains were enriched for GH29 and GH95 l-fucosidases, which could potentially cleave the terminal fucose residues that decorate mucin and human milk oligosaccharides (37). In humans, both blood ABH antigens and fucose modifications are more prevalent in ileal mucins, with potential implications for the relative localization of *Akkermansia* strains in the GI (38).

Another function with phylogroup-specific differences is the CRISPR/cas systems. While only a few of the phylogroup AmII genomes had CRISPR gene clusters, putative CRISPR/*cas* genes were detected in some AmI and in all AmIV genomes. The class of CRISPR system may be phylogroup-specific since AmIV strains were predicted to encode genes found in type I-B CRISPR/*cas* systems, while AmI strains were predicted to have genes associated with type II systems (Amuc_2008, Amuc_2009, and Amuc_2010 in Muc^T^) (39, 40), although it is not clear if these represent complete, functional systems. In some instances, the CRISPR genes are located close to predicted phage genes, possibly indicative of horizgontal genes transfer.

### Phylogroups AmII and AmIV are deficient for reductive sulfur assimilation.

**FIG 6:**
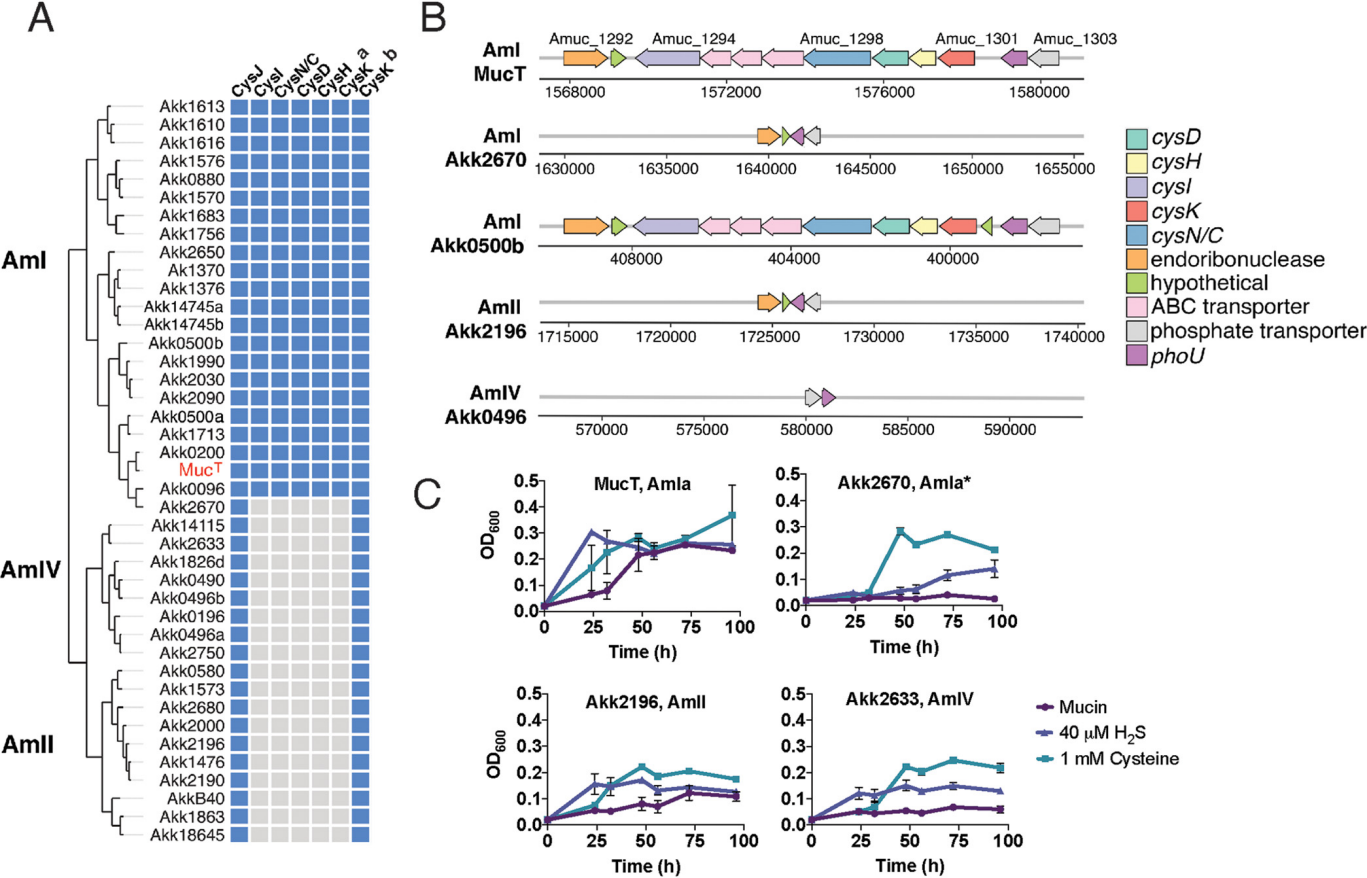
AmII and AmIV phylogroups are defective for assimilatory sulfate reduction (ASR). (A) Distribution of ASR genes among *A. muciniphila* phylogroups. ASR genes (top) from *A. muciniphila* Muc^T^ were used to search for homologs among other sequenced isolates. Blue squares indicate that a gene is present, and gray squares indicate that a gene was not detected. There are two *cysK* homologs in Muc^T^, Amuc_1301 and Amuc_2014. (B) *Genomic context of ASR genes in Akkermansia* phylogroups. The majority of the ASR genes are clustered in a single locus in the AmI phylogroup, as represented in strains Muc^T^ and Akk0500B. The AmI strain, Akk2670, lacked the entire ASR locus, although the flanking genes remained conserved in other AmI strains. The AmII strain (Akk2196) also lacked the entire ASR locus, while the AmIV strain (Akk0496) missed the locus and flanking genes. Arrows represent genes, polarity of reading frame, and the numbers above each arrow indicate the gene number in the annotated genome. The genome coordinates for each locus are shown below the arrows. (C) Addition of cysteine or NaHS enhances the growth of ASR-deficient *A. muciniphila*. Representative Am strains from each phylogroup were tested for growth in mucin medium with or without the addition of 1 mM l-cysteine or 40 μM NaHS.

Analysis of the metabolic capabilities of the strains based on genomic sequences revealed additional predicted phylogroup-specific features (Fig. 6 and Fig. S3A). For instance, metabolic enrichment analysis showed that genes required for assimilatory sulfate reduction (ASR) are significantly enriched in phylogroup AmI (adjusted *q* value, 6.14E-7) (Table S5) but absent in AmII and AmIV isolates (Fig. S3A). Enzymes in the ASR pathway reduce sulfate to hydrogen sulfide for the synthesis of sulfur-containing molecules such as cysteine and methionine. In the canonical ASR pathway, sulfate is first reduced to adenosine phosphosulfate (APS) by ATP sulfurylase (CysN), followed by the formation of phosphoadenosine phosphosulfate (PAPS) by APS kinase (CysD), which is further reduced to sulfite by PAPS reductase (CysH) and, finally, to H_2_S by sulfite reductase (CysI/J) (41). H_2_S is a substrate for cysteine synthase (CysK) to generate cysteine. In the Muc^T^ strain, ASR genes are clustered in a single locus (Amuc_1294 to Amuc_1301), with the exception of a CysJ homolog (Amuc_0631) and a second cysteine synthase (Amuc_2014). The locus also included a potential inner membrane sulfide permease (Amuc_1295), an ABC transporter-related ATP-binding protein (Amuc_1296), and a substrate-binding protein (Amuc_1297) (Fig. 6A and B).

10.1128/mBio.00478-21.5 TABLE S5 Phylogroup-specific enrichment of metabolic pathways. Download Table S5, XLSX file, 0.02 MB. Copyright © 2021 Becken et al. 2021 Becken et al. https://creativecommons.org/licenses/by/4.0/ This content is distributed under the terms of the Creative Commons Attribution 4.0 International license.

10.1128/mBio.00478-21.9 FIG S3 Genomic analysis of *A. muciniphila* strains and predicted functions. (A) Pathway enrichment analysis indicates selective acquisition and loss of metabolic pathways. To compare the metabolic capabilities of isolates, we used Anvi’o to estimate metabolism function to detect KEGG module pathways in the genomes. The lower threshold for detecting a pathway was set to 50%. Strains with 50% or less of the enzymes required for a given pathway are represented as blue squares in the heatmap. (B) Detection of selected glycoside hydrolase families by clade. Bars represent the average number of enzymes per genome. The analysis included genomes from clade AmIa (*n* = 5), AmIb (*n* = 20), AmII (*n* = 10), and AmIV = (*n* = 8). GH, glycoside hydrolase; NC, nonclassified glycoside hydrolases. Error bars represent the standard error of the mean (SEM). Download FIG S3, GIF file, 1.5 MB. Copyright © 2021 Becken et al. 2021 Becken et al. https://creativecommons.org/licenses/by/4.0/ This content is distributed under the terms of the Creative Commons Attribution 4.0 International license.

AmII and AmIV strains lacked the ASR gene cluster found in Muc^T^ but retained distally encoded homologs for CysJ and the Amuc_2014 cysteine synthase (CysK-b) (Fig. 6A). All AmI strains were predicted to perform ASR, except for the strain Akk2760 (Fig. 6A and Fig. S3A), which lacked the entire ASR locus (Fig. 6B). Since Akk2670 grew very poorly in mucin medium, with a growth rate comparable to that of AmII and AmIV isolates (Fig. 5A and Table S3), we hypothesized that the inability to generate reduced sulfur may be a limiting factor for their growth on mucin *in vitro*. To test this, representative strains were grown in mucin medium with or without the addition of cysteine or sodium hydrosulfide as a source of H_2_S. While cysteine and H_2_S shortened the lag time for the growth of the Muc^T^ strain, it did not affect the maximal biomass achieved (Fig. 6C). In contrast, cysteine significantly enhanced the maximal growth of the predicted ASR-deficient AmIa strain Akk2670, the AmII strain Akk2196, and the AmIV strain Akk0496 (Fig. 6C). These findings suggest that *A. muciniphila* strains benefit from the addition of reduced sulfur when grown in mucin and that the growth of ASR-deficient phylogroups is significantly enhanced by the addition of cysteine or H_2_S.

### AmIV strains outcompete other phylogroups in a murine colonization model.

**FIG 7:**
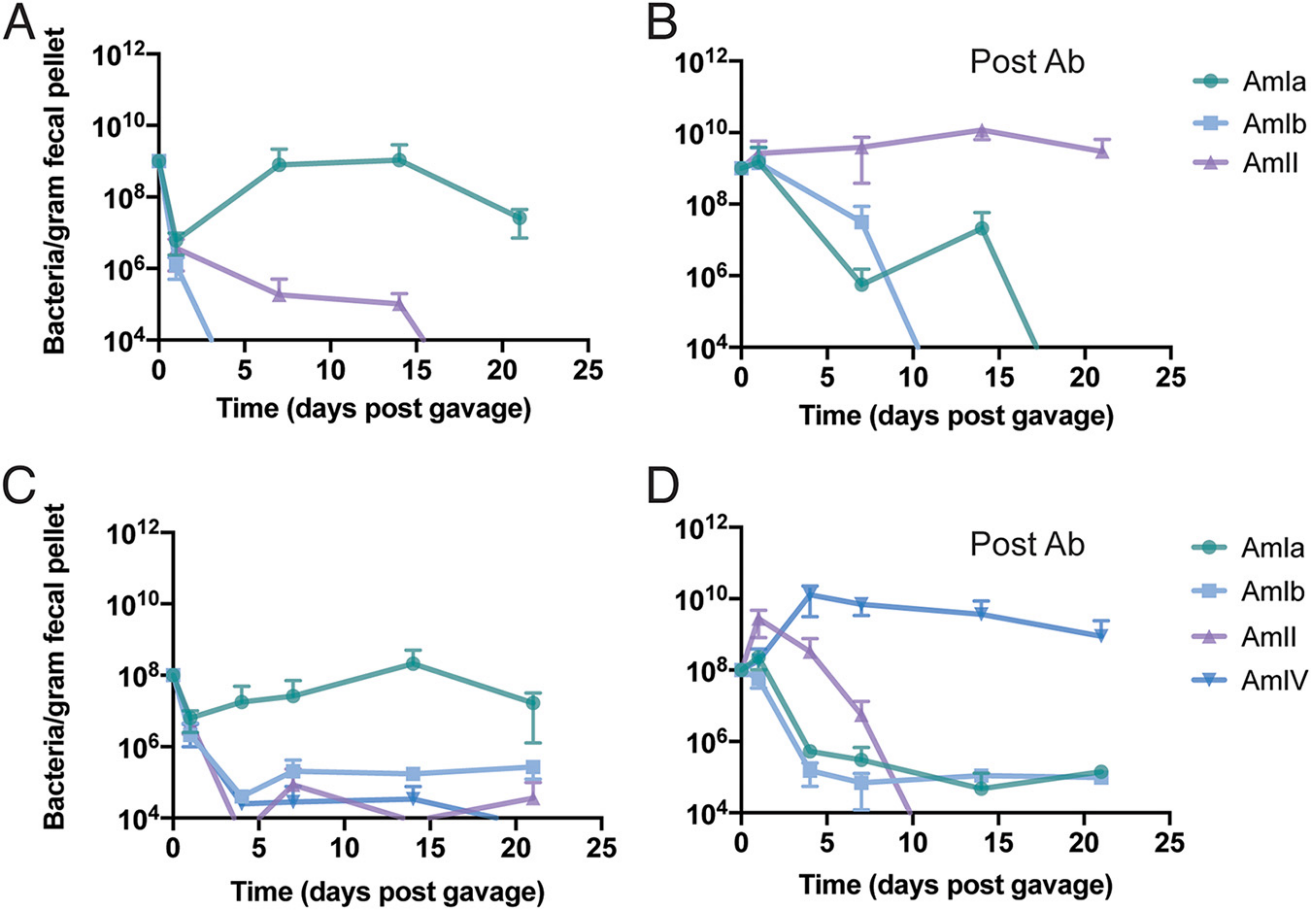
*A. muciniphila* phylogroups AmII and AmIV strains outcompete AmI in antibiotic pretreated mice. (A to D) Mice were either untreated (A and C) or treated (B and D) with antibiotics (Ab) and gavaged with a three-phylogroup strain mix containing an equal amount of phylogroups AmIa (Muc^T^), AmIb (Akk1683), and AmII (Akk0580) (A and B) or a four-phylogroup strain mix containing phylogroups AmIa (Muc^T^), AmIb (Akk1570), AmII (Akk0580), and AmIV (Akk0490) (C and D). The AmIa strain identified in mice that had not been pretreated with Ab (A and C) represents the endogenous mouse *Akkermansia*. Each point represents the average of three cages (*n *= 4 mice/cage for panels A and B, and *n* = 2 mice/cage for panels C and D), and error bars represent the standard deviation. The Am1a strain in panels A to C represent the endogenous mouse *A. muciniphila* strain found at the Duke University vivarium.

To determine if *A. muciniphila* phylogroups varied in how they colonize animals, we assessed the ability of representative isolates of each phylogroup to compete in the mouse GI tract. Mice housed in our vivarium are naturally colonized by a mouse *Akkermansia* strain, which belongs to the AmIa phylogroup (not shown). We first challenged these mice with human strains representing phylogroups AmIa, AmIb, and AmII (Fig. 7A), and their abundance in fecal pellets was monitored over time by quantitative PCR (qPCR). We found that the human AmIa, AmIb, and AmII strains failed to engraft with the endogenous mouse *A. muciniphila* and associated microbiota, which provided colonization resistance against introduction of additional *Akkermansia* strains. We cleared mice of their *Akkermansia* and other microbes with a 14-day regimen of tetracycline and repeated the competition experiments with the same cocktail of human *A. muciniphila* phylogroup representatives. Under these conditions, the AmII strain (Akk0580) became the dominant phylogroup by 20 days postinoculation (Fig. 7B).

Next, we competed strains representing all four phylogroups—AmIa, AmIb, AmII, and AmIV. As with the previous experiment, the human *A. muciniphila* isolates failed to colonize mice with an intact microbiota (Fig. 7C). However, pretreatment with antibiotics enabled engraftment of the newly introduced strains, but this time the AmIV strain (Akk0490) became the predominant phylogroup (Fig. 7D). These findings were recapitulated with a second set of representative strains, with the AmIV isolate (Akk2750) rapidly becoming the dominant phylogroup (Fig. S4). These findings suggest that AmIV strains, despite their slow growth in mucin medium and high sensitivity to oxygen, overtake other phylogroups in the GI tract when placed in direct competition in mice whose microflora had been depleted.

10.1128/mBio.00478-21.10 FIG S4 *A. muciniphila* phylogroup AmIV outcompetes other phylogroups in colonizing the mouse GI. (A) Fecal samples were collected prior to gavage, and DNA was extracted to assess endogenous *Akkermansia* levels in mice with and without antibiotic treatment. *Akkermansia* was detected with primers specific for the *Akkermansia* 16S rRNA gene (total) and with phylogroup-specific primers. (B) *In vivo* competition assay using a second set of representative strains for each phylogroup. A representative of each strain from a healthy control was selected as follows: AmIa (Akk2670), AmIb (Akk2650), AmII (2680), and AmIV (2750). Mice were treated with antibiotics until the endogenous *Akkermansia* was cleared and were subsequently gavaged with a cell suspension containing each of the phylogroups. Colonization was monitored over time by collecting fecal pellets and testing for colonization using qPCR with phylogroup-specific primers. Download FIG S4, TIF file, 2.8 MB. Copyright © 2021 Becken et al. 2021 Becken et al. https://creativecommons.org/licenses/by/4.0/ This content is distributed under the terms of the Creative Commons Attribution 4.0 International license.

### Evidence for phylogroup exclusion and switching in patients colonized with *A. muciniphila*.

**FIG 8:**
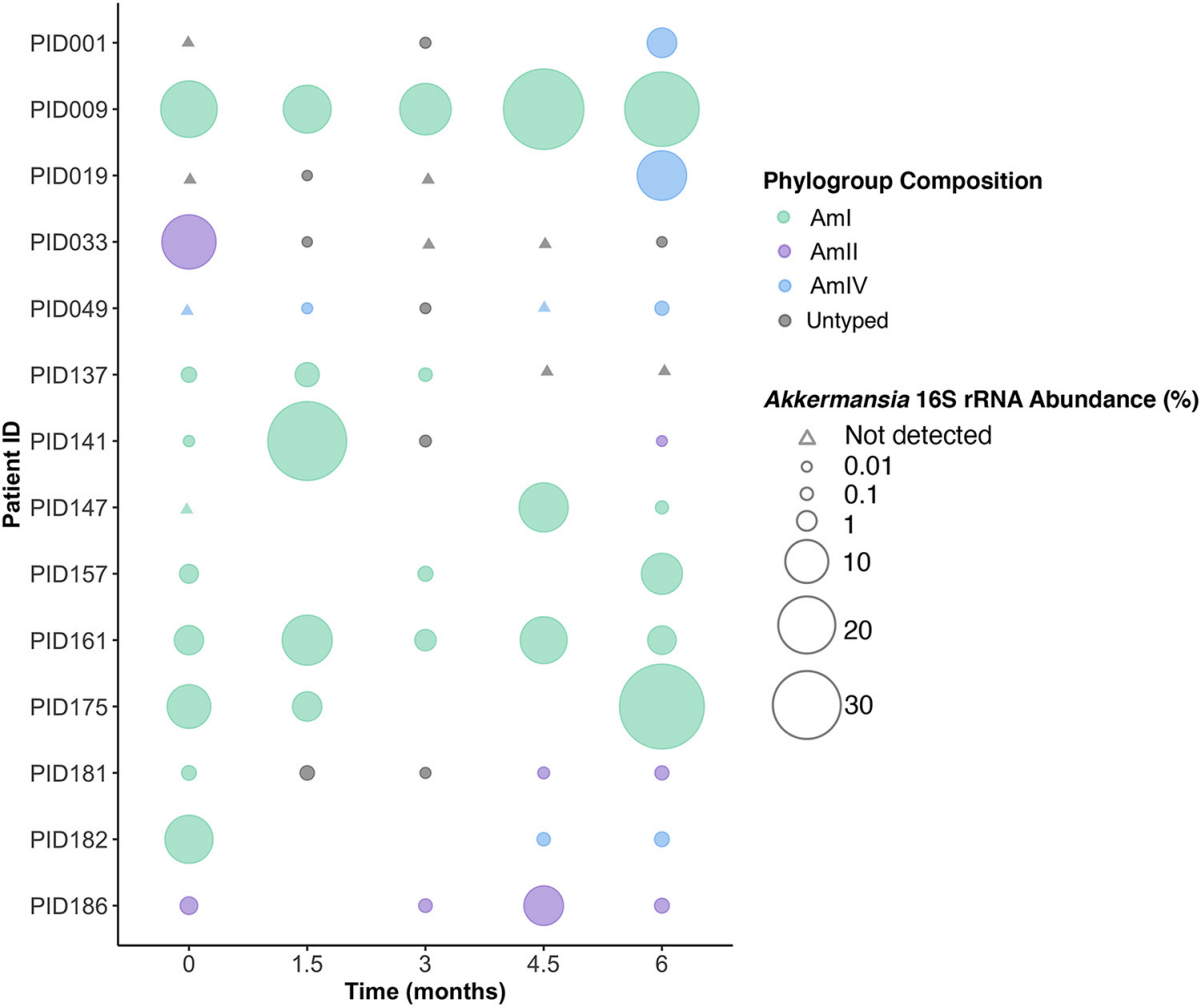
Evidence for single *A. muciniphila* phylogroup dominance in humans. A subset of stool samples from 14 patients (PID, patient ID) were selected for detection and quantification of specific phylogroups by qPCR. Samples were selected for analysis if patients had provided multiple samples (*n *> 3) throughout a 6-month period, and 16S rRNA community profiling indicated the presence of *Akkermansia* at at least one time point. The size of the bubbles represents the relative abundance of *Akkermansia* as assessed with QIIME analysis. Triangles indicate that no *Akkermansia*/*Verrucomicrobia* 16S rRNA sequences were detected. The frequency of phylogroup types was assessed by qPCR with specific primers and is color coded. In all cases, the dominant phylogroup represented >99.9% of total *Akkermansia* with a threshold for phylogroup identification set at a quantification cycle (*C_q_*) value of 34 or lower (Table S5). Missing data points indicate that no sample was collected at that time point.

Because AmII and AmIV strains prevented AmI strains from establishing themselves when placed in direct competition in mice, we hypothesized that *A. muciniphila* phylogroups occupy the same ecological niche and that AmII and AmIV strains have a selective advantage in the GI tract. To assess the natural distribution of major phylogroups in humans, we used phylogroup-specific primers (Table S4) to quantify AmI, II, and IV strains in fecal samples collected from 14 patients at baseline and at 1.5-month intervals after enrolling in POMMS (Fig. 8). In stool samples where a clade-specific signal could be detected above background, we found dominance of a single major phylogroup regardless of the total relative abundance of *Akkermansia* in that sample. Surprisingly, in three instances, the phylogroup of the *A. muciniphila* strains cultured (Akk1476, Akk1573, and Akk14115) did not match the dominant phylogroup identified by qPCR in the stool sample, and in one patient we isolated both AmII and AmIV strains (Akk1826b and Akk1826d) from the same fecal material, even though AmIV was the only strain identified by qPCR. This discrepancy between culture-based isolation and molecular quantification may reflect biases in culturing efficiency based on differences in oxygen tolerance that could impact the viability of *Akkermansia* during the collection, transport, and handling of stool samples or differences in doubling times in porcine mucin medium during the serial enrichment process. Nonetheless, these findings suggest that despite the predominance of any one *Akkermansia* phylogroup in the GI tract, additional strains can be present and viable despite being below the levels of detection by molecular methods.

In addition to evidence for the coexistence of minor clades, we observed major changes in the overall abundance of *Akkermansia* phylogroups within the same patient. In 11 of 14 of patients with multiple sampling over a 6-month period, either the relative amount of *Akkermansia* fluctuated significantly (>100-fold) among samples or the identity of the phylogroups switched (Fig. 8 and Table S6). For instance, three patients (PID141, PID181, and PID182) switched their predominant phylogroup from an AmI strain to either AmII or AmIV strains by 6 months. In two patients that underwent bariatric surgery (PID001, PID019), both converted from almost undetectable levels of *Akkermansia* at baseline to a phylogroup AmIV-dominant microbiota by the 6-month time point. It is unclear if the emergence of new dominant phylogroups represents blooms of preexisting low-abundance strains or new colonization events.

10.1128/mBio.00478-21.6 TABLE S6 Phylogroup typing in patients with obesity over a 6-month period. Download Table S6, XLSX file, 0.01 MB. Copyright © 2021 Becken et al. 2021 Becken et al. https://creativecommons.org/licenses/by/4.0/ This content is distributed under the terms of the Creative Commons Attribution 4.0 International license.

## DISCUSSION

Obesity is a multifactorial disease influenced by host genetics, diet, behavior, and the microbial ecosystems that populate the human GI tract. Diet remains a key driver of microbial composition, and the phenotypes associated with these resident microbial communities strongly influence their impact on the immunological and metabolic health of their host (42). However, some bacterial species seem to play an oversized role on the health of their hosts. For instance, *A. muciniphila* has emerged as a potential probiotic since its abundance in the GI tract positively correlates with decreased incidence of metabolic disease, obesity, and diabetes (9, 10).

There is a growing recognition that there is a great diversity of *Akkermansia* strains and species. A pangenomic analysis of *Akkermansia* genomes revealed four *A. muciniphila* phylogroups (16), and an analysis of >1,000 *Akkermansia* genomes reconstructed from metagenomic sequences from human samples across the world suggested the existence of up to four new species (43, 44). While metabolic capacities of different *Akkermansia* strains have been inferred based on genome annotations, experimental validation is largely lacking because isolates of these new phylogroups have either not been collected or not been characterized. A notable exception is a recent analysis of the vitamin B_12_ biosynthetic properties of a member of the AmII phylogroup (16).

In this work, we leveraged fecal samples collected as part of the POMMS interventional study for childhood obesity (18) to isolate *Akkermansia* strains that reflect the diversity of phylogroups present in both healthy and diseased states in children. Importantly, the availability of cultured strains enabled the phenotyping of each isolate to identify variances in traits that may impact the health of their hosts. We cultured mucophilic bacteria from 123 fecal samples derived from 49 donors and isolated 71 new strains of *A. muciniphila* (Table S1 and S3). The relative abundance of *Verrucomicrobia* amplified sequence variants (ASVs) ranged from nondetectable to >30% of total sequences in children with obesity (Table S2).

Based on the genomes of 43 of new *Akkermansia* strains, we determined that these isolates belonged to *A. muciniphila* phylogroups AmI, II, and IV (Fig. 4). AmI strains constituted over half of the isolates, which we propose should be subdivided into two subgroups (AmIa and Ib) based on an ANI cutoff of 96% among complete genome sequences and phylogroup-specific phenotypes, such as increased sensitivity to ambient O_2_ for AmIb strains (Fig. 5A). In contrast, AmII strains were more resistant to ambient O_2_ than all the other phylogroups. AmII strains were also more immunostimulatory for TLR2 and TLR4, although this was most apparent when bacteria were grown in mucin, as opposed to synthetic medium (Fig. 5F and G and Fig. S2). AmIV strains also displayed enhanced activation of TLR4 and TLR2 reporter cell lines, which may reflect their increased binding to epithelial cells (Fig. 5C and D). Using BMDM, we confirmed that TLR2 and TLR4 are the relevant pattern recognition receptors for the detection of *A. muciniphila* as previously reported (23), with TLR2 activation requiring a higher threshold for activation than that of TLR4 (Fig. 4A). TLR2-mediated enhancement of tight junctions in intestinal epithelia has been proposed as a mechanism to explain *Akkermansia*-dependent enhancement of gut barrier function (23). TLR2 forms heterodimers with TLR1 or TLR6, with TLR2/1 being preferentially activated by triacylated lipopeptides from Gram-negative bacteria, and TLR2/6, by diacylated lipopeptides most commonly expressed on Gram-positive bacteria (45, 46). Amuc_1100, a highly expressed pilus-like protein conserved among all phylogroups, has been proposed to be a major *A. muciniphila* TLR2 agonist (14, 23). We suspect additional TLR2 agonists exist, given that *A. muciniphila* can stimulate HEK2923 cells expressing either TLR2/1 or TLR2/6 alone.

Although *A. muciniphila* is classified as a strict anaerobe, the growth of the reference Muc^T^ (AmIa) isolate is enhanced by nanomolar concentrations of O_2_ (22). The ability to use oxygen as a terminal electron acceptor may provide *A. muciniphila* an advantage over other colonic bacteria when in proximity to epithelial surfaces. The closely related AmIb strains, however, are very sensitive to ambient oxygen even though the genomes are highly similar and also encode the cytochrome *bd* complex, which is required to use O_2_ as a terminal electron acceptor (22). The mechanism underlying this differential sensitivity to oxygen is not apparent from comparative genomics. In contrast, AmII strains’ high resistance to oxygen may be linked to ferrous and ferric iron transport, as genes encoding Fe^3+^ ABC transporters have expanded in AmII strains and iron transporter genes are highly expressed in *A. muciniphila* Muc^T^ when switched to aerated growth conditions (22). AmIV strains also display high sensitivity to oxygen, which may also be associated with decreased iron acquisition, as this phylogroup lacks components of the enterochelin transport system present in AmI strains.

Given the slow doubling times for both AmII and AmIV strains *in vitro*, we did not expect these strains to overtake AmI strains in antibiotic-treated mice. One possible explanation is that the overrepresentation of capsular and exopolysaccharide gene clusters in AmII and AmIV phylogroups may provide protection from IgA and host-derived antimicrobial peptides. Unexpectedly, AmIV strains outcompeted AmII strains in mice, even though AmIV was expected to be more sensitive to the oxygen present near colonic epithelium. Given these findings, it is clear that the complexity of the GI ecosystem makes it difficult to predict which *in vitro* phenotypes are most relevant for *in vivo* colonization of the GI tract.

A comparison of the predicted metabolic pathways in *A. muciniphila* strains indicated a clear absence of components of the assimilatory sulfate reduction (ASR) pathway in AmII and AmIV strains and in one AmIa isolate (Akk2670) (Fig. 6). ASR is required to harvest sulfur from imported sulfate for the biosynthesis of amino acids. Akk2670 was unique among AmI strains in that it displayed very slow growth rates in gastric mucin. This led us to postulate that the long doubling times for Akk2670, and AmII and AmIV isolates, reflect a reduced capacity to synthesize sulfur-containing amino acids. Although mucin contains sulfur in the form of cysteine and methionine in the protein backbone and terminally sulfated glycans (47), the cysteine and methionine content may not be sufficient to support rapid growth, and sulfate cannot be used in the absence of a functional ASR pathway. The ASR pathway generates H_2_S, which is used to synthesize cysteine through condensation with *O*-acetylserine by cysteine synthase (48). Most AmI strains have two putative cysteine synthases (Amuc_1301 and Amuc_2014 in Muc^T^), with Amuc_2014 being conserved in all *Akkermansia* (Fig. 6B). Cysteine also plays an essential role as a sulfur source for the biosynthesis of essential cofactors, vitamins, and antioxidants (48). Consistent with this prediction, the growth of Akk2670, AmII, and AmIV strains is significantly enhanced by the addition of exogenous cysteine or H_2_S to mucin medium (Fig. 6C).

It is clear that the loss of ASR is not essential for GI colonization by *A. muciniphila* and may even enhance competitiveness given that sulfate transport and reduction is an energy intensive process (49, 50), particularly if other sources of reduced sulfur are available from the host or the microbiota. Potential sources of reduced sulfur in the GI tract include taurine, low levels of cysteine, and H_2_S (51). For some members of the *Bifidobacterium* genus, cysteine auxotrophies are prominent, and at least in the case of B. bifidum, cysteine auxotrophy cannot be rescued by supplementation of glutathione or taurine (52). Finally, several bacterial pathogens are cysteine auxotrophs, and even among species with complete ASR systems, clinical isolates have been observed to spontaneously become cysteine auxotrophs (48). Thus, there may be selective pressure for the loss of ASR genes in the presence of alternative reduced sulfur sources.

The loss of ASR in some *A. muciniphila* strains suggests that AmII and IV strains could be net consumers of any microbiota-derived H_2_S, especially under conditions where AmII and AmIV strains constitute a significant proportion of the entire microbiota (Fig. 8). If so, their localized detoxification of H_2_S may contribute to some of the protection that has been ascribed to *Akkermansia* in the context of inflammatory bowel disease (IBD) and Crohn’s disease (53–55). On the other hand, H_2_S derived from the breakdown of cysteine by intestinal cystathionine β-synthase has anti-inflammatory properties (56) and may stimulate the production of mucins (57). Under these circumstances, colonization by AmII and AmIV strains may be proinflammatory if they decrease the effective concentration of H_2_S at epithelial surfaces.

The relative competitive advantage of AmII and AmIV strains in antibiotic-treated mice was unexpected given the relative high prevalence of AmI strains in human populations (16, 44). However, we noted that microbiota of specific-pathogen-free (SPF) mice, which has an endogenous mouse AmI strain, provided colonization resistance, which led us to ask if a similar phylogroup exclusion is observed in humans. In stool samples collected at various time points after various interventions aimed at reducing obesity, we observed dominance by a single phylogroup. In some instances, both 16S rRNA-based community profiling and qPCR indicated that *A. muciniphila* was not detectable in baseline samples yet appeared by 6 months (PID001 and PID009), and in others, the dominant phylogroup disappeared within 3 months after baseline sample collection (PID033 and PID137), and yet in other patient samples, a phylogroup would disappear and return (PID049) or be replaced by a new phylogroup (PID141, PID181, and PID182). The abrupt disappearance of *Akkermansia* has been previously documented in densely sampled individuals (58). At this, stage we cannot distinguish between population crashes that are followed by repopulation by a newly acquired *Akkermansia* phylogroup or blooms of preexisting phylogroups that were present below the limits of detection. Evidence for the latter is supported by our ability to culture *A. muciniphila* strains that did not belong to the predominant phylogroup within the stool sample. Overall, patients are dominated by a single major phylogroup at any one time, but the abundance and identity of each *A. muciniphila* phylogroup is subject to fluctuation by environmental factors and ecological pressures that are still unknown.

The phenotypic diversity of the *A. muciniphila* strains in this cohort of patients suggests that experimental approaches using cultured strains will be critical to understand *Akkermansia* physiology. A recent survey of more than 10,000 adults in the American Gut Project established a weak inverse correlation between *A. muciniphila* abundance and BMI, with a protective role against obesity when adjusted for confounders such as sex, age, and diet (11). It is plausible that these correlations may be further strengthened when stratified by what is the most prevalent *A. muciniphila* phylogroup in an individual. It is certainly possible that while some strains are beneficial, others may be neutral or even potentially harmful (59). Even strains that are considered beneficial, such as Muc^T^, may be harmful in the “wrong” context depending on the host’s inflammatory status, diet, or microbiota (60). The observation that patients can be colonized by different strains at different times suggests that *A. muciniphila* colonization is a dynamic process, especially considering that its primary food source, host mucins, should not be subject to the same variability as the diet-derived carbohydrates used by other intestinal microbes. Determining which *A. muciniphila* strains are most beneficial, and what factors influence strain-specific colonization, will be critical for the development of effective *A. muciniphila*-based probiotics.

## MATERIALS AND METHODS

### Media, strains, and growth conditions.

Bacteria were isolated and grown in an anaerobic chamber (Coy Laboratory) with the following gaseous characteristics: 5% hydrogen, 5% carbon dioxide, and 90% nitrogen. *A. muciniphila* was grown in mucin medium based on previous work (7) (3 mM KH2PO4, 3 mM Na_2_PO_4_, 5.6 mM NH_4_Cl, 1 mM MgCl_2_, 1 mM Na_2_S · 9H_2_O, 47 mM NaHCO_3_, 1 mM CaCl_2_, and 40 mM HCl, trace elements and vitamins [61], and 0.25% porcine gastric mucin [type III, Sigma-Aldrich]). Additional media used to culture *Akkermansia* included synthetic media, where porcine gastric mucin was replaced with 0.2% GlcNAc, 0.2% glucose, 16g/liter of soy peptone and 4 g of threonine/liter (14), and BD Bacto brain heart infusion broth (BD; catalog number 237500) with 0.25% porcine gastric mucin. To test growth with cysteine, mucin medium was supplemented with filter-sterilized l-cysteine to a final concentration of 0.5 mM. The *A. muciniphila* strain Muc^T^ (7) was obtained from ATCC (BAA-835). HEK-Blue hTLR2/1/6 and hTLR4 were obtained from InvivoGen (hkb-htlr2, hkb-htlr4) and maintained as described by the manufacturer. HT29-MTX was from Sigma (12040401-1VL) and maintained in Dulbecco’s modified Eagle medium (DMEM) (Gibco 11995-065) supplemented with 10% fetal bovine serum.

### Isolation of *A. muciniphila* from fecal samples.

The recruitment criteria and composition of stool donors in the POMMS Study were previously reported (18). Approximately 75 mg of frozen stool was used to inoculate 1 ml of mucin medium supplemented with vancomycin (6 μg/ml), gentamicin (10 μg/ml), and kanamycin (12 μg/ml) and incubated at 37°C for 48 h. After three sequential passages in mucin medium, a sample of the suspension was streaked on 1% agar mucin medium plates to isolate single colonies and was incubated for 7 days at 37°C. Colonies of unique morphology were restreaked on 1% agar BBL brain heart infusion (BD Biosciences; catalog 211065) plates supplemented with 0.2% mucin and incubated for 4 days. Total DNA was isolated, and the strain was identified by PCR-based amplification of the 16S rRNA gene V3-V4 region. The nomenclature used for new *A. muciniphila* strains is as follows: Akk*XXXYn*, with *X* being the patient number (009-275), *Y* the month of sample collection, (0,1.5,3,4.5, or 6) and *n* the clone typed if more that one colony was collected per plate (*a* to *d*; no letter indicates that only one colony was picked for analysis). AkkB40 is an isolate from a healthy adult male.

### Global analysis of microbial composition by 16S rRNA sequencing.

The composition of total bacteria in stool samples was determined from DNA samples extracted from stool with a Qiagen stool extraction kit (Qiagen; catalog number 51604) by amplification of the 16S rRNA gene by PCR using primers 515 and 806 as described in the Earth Microbiome Project protocols, followed by DNA sequencing of the resulting amplicons on an Illumina MiSeq platform.

Phylogenetic analysis was performed using the Quantitative Insights into Microbial Ecology 2 (QIIME 2) platform version 2019.7 (62). Raw sequence data were demultiplexed using the emp-paired option (63, 64), followed by denoising with DADA2 (65) using the parameters p-trim-left-f 10, p-trim-left-r 10, p-trunc-len-f 233, and p-trunc-len-r 164. Amplicon sequence variants (ASVs) were assigned taxonomy using the feature classifier classify-sklearn (66, 67) and the SILVA 132 99% 515F/806R reference sequences (67).

### Phenotypic characterization of *A. muciniphila* isolates.

**Growth rate determination.** Growth rates were assessed in both liquid mucin and synthetic medium. Starter cultures were grown to saturation in 3 ml synthetic medium supplemented with 0.25% porcine gastric mucin and diluted 1:5 into fresh medium and grown for an additional 8 h. The resulting cultures were then diluted 1:25 into fresh medium (optical density at 600 nm [OD_600_], 0.01 to 0.05), and 150-μl aliquots were dispensed into 96-well microplates. Each well was covered with 100 μl of paraffin oil and incubated at 37°C in a BMG SpectroStar Nano plate reader under anaerobic conditions. The optical density (OD_600_) was measured at 1-h intervals for 72 h. Generation times and growth rates were determined using the R package Growthcurver (version 0.3.0) (68). Results were obtained from three biological replicates per strain.

**Growth with cysteine and sodium hydrogen sulfide**. Growth was tested in medium supplemented with l-cysteine or the H_2_S donor sodium hydrogen sulfide (NaSH) (Cayman Chemical; catalog number 10012555). NaSH stock solutions were prepared in phosphate-buffered saline (PBS) under anaerobic conditions. *Akkermansia* starter cultures were standardized to an OD_600_ of 0.5 and diluted 1:25 into 3 ml of mucin medium with or without 1 mM l-cysteine or 40 μM NaSH. The cultures were incubated anaerobically at 37°C, and the optical density was measured over 4 days. All assays were run in triplicate.

**Agglutination**. Actively growing cultures were used to inoculate 1.2 ml of mucin medium in fresh deep 96-well plates and incubated anaerobically at 37°C for 3 days. The degree of bacterial sedimentation was quantified by removing 150 μl of culture from the top of the well. Agglutination was calculated for each strain as:

$$\text{agglutination}=1-(\frac{\text{mean OD}600\text{ of TOP}}{\text{mean OD}600\text{ of WHOLE}})$$

Three biological replicates of this assay were performed for every strain, and each contained three technical replicates.

**Adherence to epithelial cells**. HT29-MTX cells were seeded into 96-well plates at a density of 2.5 × 10^4^ cells per well and grown for 7 days past confluence. Wells were washed twice with PBS and incubated with 2.5 × 10^6^ *A. muciniphila* cells in DMEM for 2 h at 37°C under anaerobic conditions. As a control for nonspecific binding of *Akkermansia*, UltraCruz high binding enzyme-linked immunosorbent assay (ELISA) (sc-204463) plates were precoated with 100 μl of 1% bovine serum albumin (BSA). Wells with HT29-MTX cells or coated with BSA were washed twice with PBS to remove nonadherent bacteria. Synthetic medium (100 μl) was added to each well, and plates were cultured for either 48 h or 96 h at 37°C under anaerobic conditions. HT29-MTX cell or BSA binding was assessed by measuring the optical density at 600 nm after outgrowth in the assay wells and calculating the ratio of HT29-MTX coated OD:BSA coated OD. Data are reported as the average and standard deviation of 3 technical replicates from 3 to 4 independent biological replicates. For microscopy, HT29-MTX cells were seeded into 24-well plates with 12-mm round glass coverslips at a density of 1 × 10^5^ cells per well and grown for 7 days past confluence. Wells were washed twice with PBS and incubated with 1 × 10^6^ *A. muciniphila* cells in 500 ml anaerobic-adapted DMEM for 2 h at 37°C under anaerobic conditions. Wells were washed twice, fixed with 3.7% formaldehyde in PBS for 30 min on ice, washed twice with PBS, and blocked overnight at 4°C in blocking buffer (2% [wt/vol] BSA in PBS). Coverslips were incubated with a 1:50 dilution of anti-*Akkermansia* polyclonal antibody followed by an incubation with goat anti-rabbit-488 (Invitrogen; catalog number A-11008) and Hoechst for 1 h at 25°C. After two washes, coverslips were mounted on slides with Vectashield medium and imaged on a Nikon Eclipse Ti2 inverted microscope with ×20 objective.

**Measurement of short-chain fatty acids (SCFA).** For each strain, 1 ml of culture supernatants from strains grown in mucin medium was removed for SCFA analysis following the protocol of Holmes et al. (69). In brief, the supernatant was centrifuged at 14,000 relative centrifugal force (rcf) for 5 min at 4°C to pellet debris, and then 750 μl of supernatant was passed through a 0.22-mm spin column filter. The resultant filtrate was then acidified to a pH of <3 with 50 μl of 6N HCL and transferred to a glass autosampler vial for analysis. Filtrates were analyzed on an Agilent 7890 gas chromatograph (GC) equipped with a flame-ionization detector (FID) and an Agilent HP-FFAP free fatty-acid column (69). The concentrations of acetate and propionate in the samples were determined using an 8-point standard curve (0.1 mM to 16 mM).

**Sensitivity to ambient oxygen**. Strains were grown from frozen stocks in 0.5 ml mucin (0.4%) medium in deep 96-well plates to saturation. After subculturing in mucin medium for 5 h, serial dilutions of each strain were spotted on BBL BHI agar (BD; catalog number 211065) plates supplemented with 0.4% mucin. One plate was left in the anaerobic chamber, while the others were exposed to ambient O_2_ for 12, 18, or 24 h, before being returned to the chamber. The relative sensitivity to ambient oxygen was determined by monitoring the ratio of CFU with and without exposure to ambient oxygen.

**Bone marrow macrophage (BMM) stimulations**. BMMs were obtained from 6- to 12-week-old C57BL/6J mice of the following genotypes: wild-type, *Tlr2*^–/–^ *Tlr4*^–/–^ *Unc93b1^3d/3d^*, *Tlr2*^–/–^ *Tlr4*^–/–^, *Tlr2*^–/–^ *Unc93b1*^3d/3d^, and *Tlr4*^–/–^ (25). Bone marrow was dissociated through a 70-μm filter, treated with ACK lysis buffer (Gibco; catalog numberA1049201), and differentiated for 6 days in DMEM complete medium (DMEM supplemented with 10% [vol/vol] fetal bovine serum, l-glutamine, penicillin-streptomycin, sodium pyruvate, HEPES, and 2-mercaptoethanol) supplemented with 10% (vol/vol) of supernatants from 3T3-CSF cells, overproducing macrophage colony-stimulating factor. For stimulations, BMMs were plated in DMEM complete medium supplemented with 10% (vol/vol) M-CSF and incubated with *A. muciniphila* at the indicated multiplicity of “infection” (MOI), 1 μM CpG-B (InvivoGen; tlr1-1668-1), 500 ng/ml Pam3CSK4 (InvivoGen; tlr1-pms), or 50 ng/ml lipopolysaccharide (LPS) (InvivoGen; tlr1-3pelps). For analysis of secreted cytokines, the supernatant was collected 4 h after stimulation and analyzed with the BD cytometric bead array mouse inflammation kit (BD Biosciences; catalog number 552364) according to the manufacturer’s instructions.

**Activation of hTLRs**. HEK-Blue hTLR2/1/6- and hTLR4-expressing cells (InvivoGen; hkb-htlr2, hkb-htlr4) were seeded into 96-well plates pretreated with poly-l-lysine. *A. muciniphila* isolates were added to each well at an MOI of 5 in triplicate. Negative controls included culture medium with 10% heat-inactivated FBS. Positive controls included ultrapure lipopolysaccharide from Escherichia coli 055:B5 at 100 ng/ml and 1 ng/ml. For the experiments using hTLR2/1/6, Pam3CSK4, a synthetic triacylated lipopeptide, was used at concentrations of 100 ng/ml and 5 ng/ml. After a 16-h incubation, levels of sAP were assessed with Quanti-Blue detection medium as detailed by the manufacturer.

### Genomic sequencing, annotation, and comparative analysis.

*A. muciniphila* genomic DNA was extracted using a MagAttract high-molecular-weight (HMW) DNA kit (Qiagen; catalog number 67563) according to the manufacturer’s protocol. The extracted DNA was ethanol precipitated, and the final concentration was determined with a Qubit double-stranded DNA (dsDNA) high-sensitivity (HS) kit (Thermo Scientific). Libraries were generated using a SMRTbell template prep kit version 2.0 (Pacific Biosciences [PacBio]) and sequenced on a PacBio Sequel instrument.

After sequencing, PacBio SMRTLink software (version 8.0.0) was used to demultiplex the samples, and the resulting BAM files were converted to fasta files using SAMtools (70). Genome assembly was performed with Flye version 2.7 with the following parameters: –genome-size 3m, –plasmids, and –meta (71). The assembled, circular genomes were then rotated to set the starting position to the *dnaA* gene using the fixstart function in Circlator (72). Finally, assembly annotation and quality evaluation were run using the PATRIC RASTtk-enabled genome annotation service (73). Sequences have been deposited in GenBank (NCBI BioProject accession number PRJNA715455.

Comparative analysis of the assembled genomes was run using the pangenomic workflow in Anvi’o version 6.2 (74, 75). First, the 43 assembly fasta files were reformatted into an Anvi’o-compatible contig database by running the script anvi-script-FASTA-to-contigs-db. This command uses Prodigal to identify open reading frames (76). The resulting databases were annotated with the script anvi-run-ncbi-cogs and subsequently combined to make a genome database using anvi-gen-genomes-storage. To compute the pangenome, the command anvi-pan-genome was run with the following parameters: –minbit 0.5, –mcl-inflation 10, and –use-ncbi-blast.

We computed average nucleotide identity (ANI) across the genomes using the command anvi-compute-genome-similarity with the –method pyani parameter (77). We included publicly available *Akkermansia* genomes as controls in our ANI and pangenome analyses. Representatives of phylogroups AmI, AmII, and AmIII were described in Guo et al. and retrieved from NCBI (17) (GenBank assembly accession numbers GCA_002885425.1, GCA_002885025.1, GCA_002884975.1, GCA_002884915.1, and GCA_002885515.1). The phylogroup AmIV representative genome CDI-150b was obtained from the JGI IMG database (16). Based on the resulting analysis, each genome was assigned to a phylogroup using the function anvi-import-misc-data, and phylogroup-specific gene functions were then obtained using the command anvi-get-enriched-functions-per-pan-group to identify cluster of orthologous groups of proteins (COGs). COGs present in all members of a given phylogroup, and absent in all other phylogroups, were considered to represent phylogroup-specific gene functions. The adjusted *q* value represents the false-discovery rate adjusted *P* value corrected for multiple testing as calculated by Anvi-o. Finally, the genomes were analyzed for metabolic pathways in Anvi’o (https://merenlab.org/software/anvio/help/main/programs/anvi-estimate-metabolism/). Each genome was annotated with the KEGG KOfam database using the program anvi-run-kegg-kofams (78, 79). The annotated genomes were then used as input to the program anvi-estimate-metabolism, with the flag –module-completion-threshold set to identify pathways with a minimum of 50% completion in at least one isolate genome. The module output was further analyzed with the command anvi-compute-functional-enrichment to test for phylogroup-specific enrichment. To visualize the data, heatmaps were generated using the pheatmap R package (80), and genes were plotted with the gggenes R package (81) in ggplot2 (82). To search for specific ASR genes among the isolates, custom BLAST databases were generated using the annotated isolate genomes (83). Searches were conducted using the sequences for the ASR genes from *A. muciniphila* Muc^T^ as the query.

To identify glycoside hydrolase families in the sequenced strains, we used dbCAN2 version 2.0.11 to annotate carbohydrate-active enzymes (84). DNA fasta files were used as input, and the annotation was run using the standalone tool run_dbcan. The resulting annotation tables were analyzed to determine the number of each type of glycoside hydrolase family per genome. Annotations detected with Diamond and at least one additional method, Hotpep or Hmmer, were considered positive.

### Mouse colonization and phylogroup competitions.

To prepare the inoculum for competition experiments, *A. muciniphila* cultures were standardized by optical density, combined using equal parts of each phylogroup to be tested, and stored at −80°C in PBS containing 20% glycerol.

All mouse experiments were approved by Duke University’s Institutional Animal Care and Use Committee. *In vivo* competition experiments were caried out using 6-week-old female C57BL/6J mice obtained from Jackson Laboratories. *A. muciniphila* colonization was tested in mice both with and without pretreatment with antibiotics (3 g/liter tetracycline suspended in distilled water with 10% sucrose for 2 weeks). Following antibiotic treatment, clearance of residual mouse *Akkermansia* was determined by PCR using *Akkermansia-*specific 16S rRNA primers (12).

For *in vivo* competition assays, mice were inoculated by intragastric gavage with a mixture containing 2.5 × 10^8^ CFU of each phylogroup in a total volume of 140 μl. The three-phylogroup competition experiment used a mixture of the strains Muc^T^ (AmIa), Akk1683 (AmIb), and Akk0580 (AmII). Two additional competition experiments were run, each using a combination of four clades. The first four-clade competition used a mixture of strains Muc^T^ (AmIa), Akk1570 (AmIb), Akk0580 (AmII), and Akk0490 (AmIV). The second four-clade competition used a mixture of strains Akk2670 (AmIa), Akk2650 (AmIb), Akk2680 (AmII), and Akk2750 (AmIV).

### Analysis of *A. muciniphila* phylogroup distribution in fecal samples from mice and humans.

Phylogroup abundance was assessed by qPCR with phylogroup-specific primers. PCR was run with PowerUp SYBR master mix reagent on a QuantStudio 3 real-time PCR system (Applied Biosystems) using fast cycling mode. The abundance of *Akkermansia* was calculated as copies per gram fecal material. For human samples, the same DNA used for 16S rDNA sequencing was used as the template for qPCR with phylogroup-specific primers (Table S4).

Unless otherwise noted, statistical analyses and plots were generated with GraphPad Prism version 9.0.0.
