# Supplementary figures and images for: Genotypic and Phenotypic Diversity among Human Isolates of Akkermansia muciniphila
Source: mBio. 2021 May 18;12(3):e00478-21. doi: 10.1128/mBio.00478-21 (PMC8262928; doi:10.1128/mBio.00478-21)

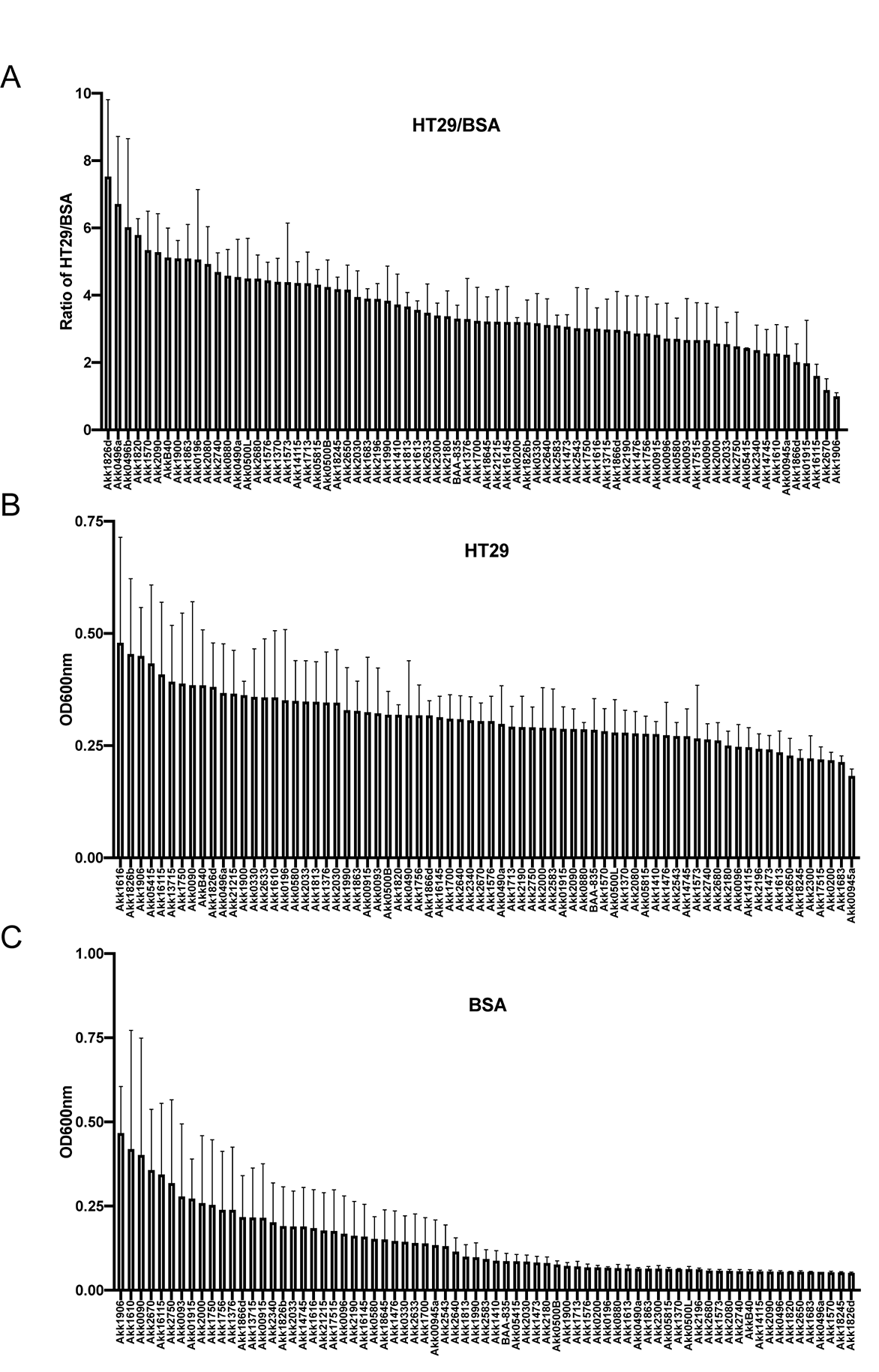

Supplement: FIG S1 [file mbio.00478-21-sf001.tif]

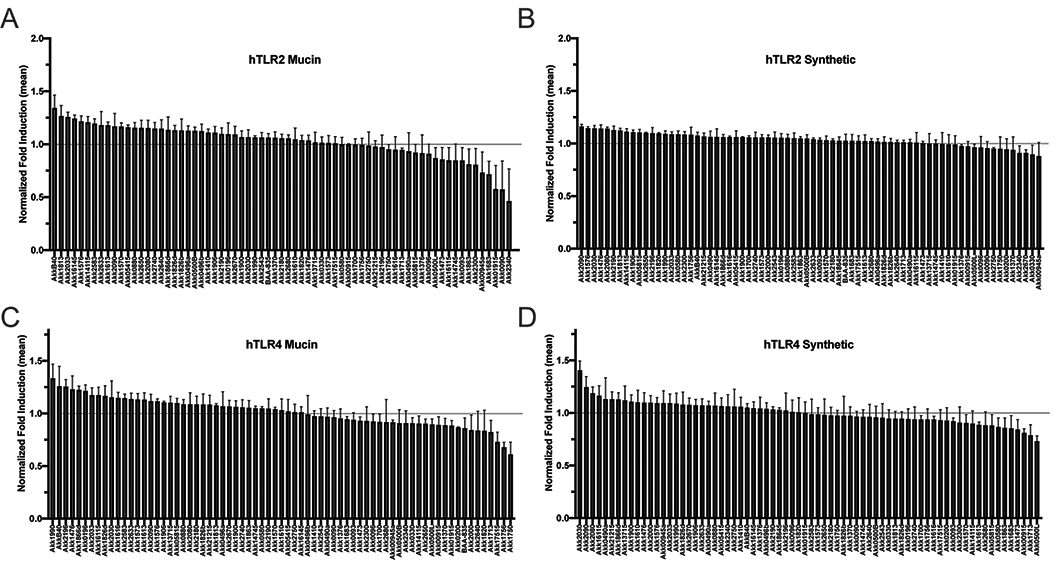

Supplement: FIG S2 [file mbio.00478-21-sf002.tif]

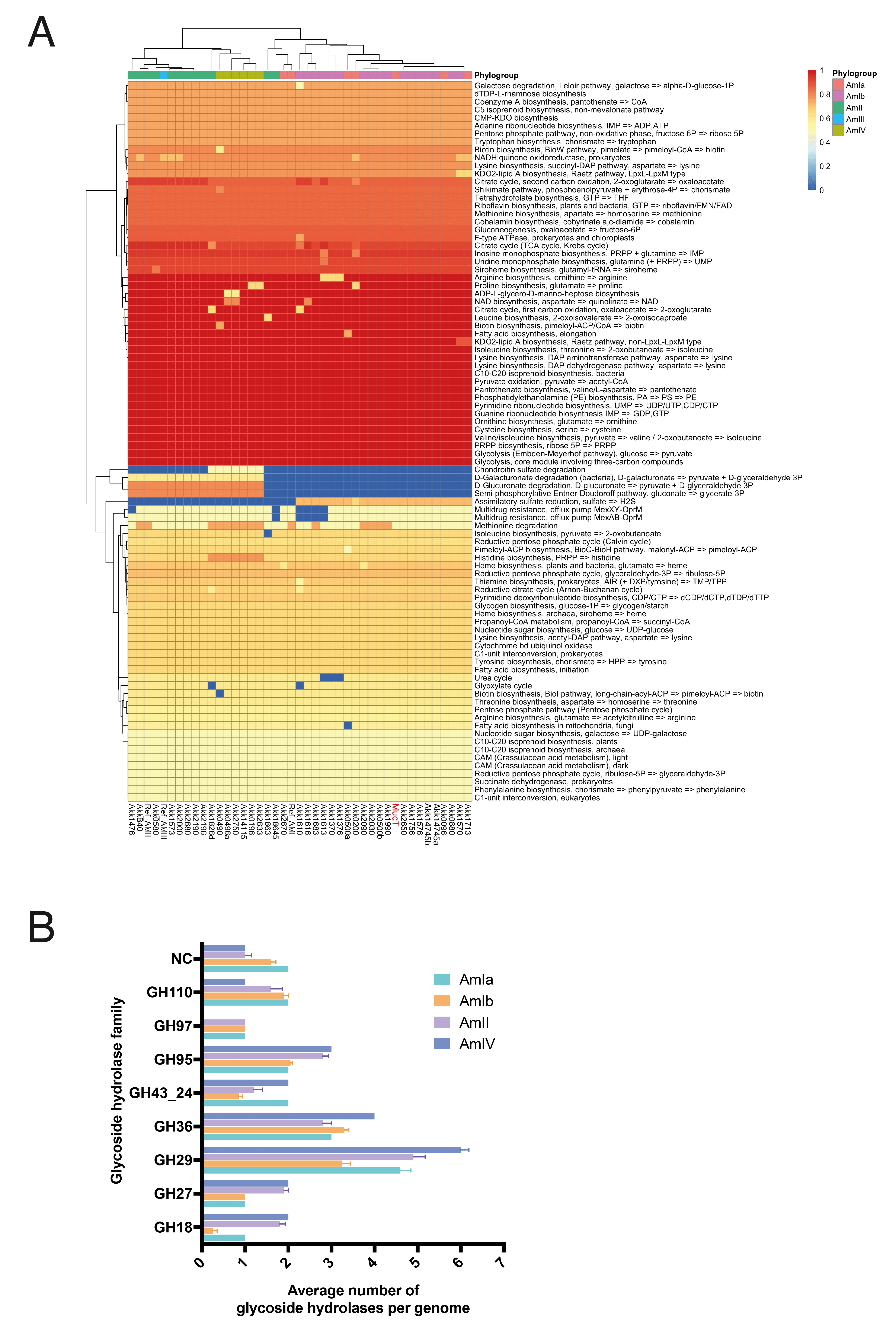

Supplement: FIG S3 [file mbio.00478-21-sf003.gif]

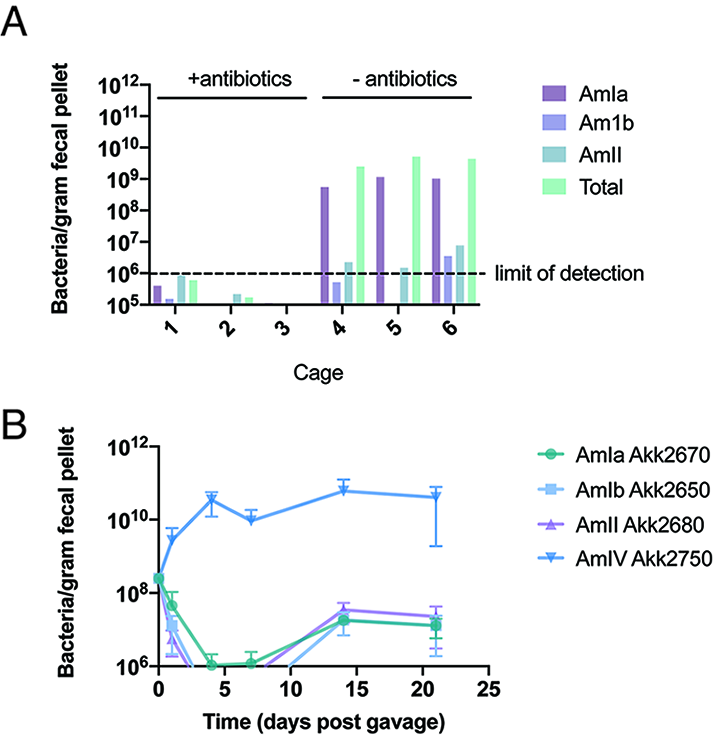

Supplement: FIG S4 [file mbio.00478-21-sf004.tif]
